# Differential tumor immune microenvironment coupled with tumor progression or tumor eradication in HPV-antigen expressing squamous cell carcinoma (SCC) models

**DOI:** 10.3389/fimmu.2024.1405318

**Published:** 2024-07-11

**Authors:** Arpitha H. Shivarudrappa, Jessy John, Monika Vashisht, Huaibin Ge, Silvia Liu, Jingxin Chen, Karen Siddoway, Rui Dong, Zhangguo Chen, Jing H. Wang

**Affiliations:** ^1^ University of Pittsburgh Medical Center UPMC Hillman Cancer Center, Division of Hematology and Oncology, Department of Medicine, University of Pittsburgh School of Medicine, Pittsburgh, PA, United States; ^2^ Department of Pathology, University of Pittsburgh School of Medicine, Pittsburgh, PA, United States; ^3^ School of Medicine, Tsinghua University, Beijing, China; ^4^ Department of Immunology, University of Pittsburgh School of Medicine, Pittsburgh, PA, United States

**Keywords:** immunological heterogeneity, head and neck squamous cell carcinoma, individualized anti-tumor immune responses, tumor immune microenvironment, T cell receptor

## Abstract

Human papilloma virus (HPV) is an etiological factor of head and neck squamous cell carcinoma (HNSCC). To investigate the role of HPV antigen in anti-tumor immunity, we established mouse models by expressing HPV16 E6 and E7 in a SCC tumor cell line. We obtained two HPV antigen-expressing clones (C-225 and C-100) transplantable into C57BL/6 recipients. We found that C-225 elicited complete eradication in C57BL/6 mice (eradicated), whereas C-100 grew progressively (growing). We examined immune tumor microenvironment (TME) using flow cytometry and found that eradicated or growing tumors exhibited differential immune profiles that may influence the outcome of anti-tumor immunity. Surprisingly, the percentage of CD8 and CD4 tumor-infiltrating lymphocytes (TILs) was much higher in growing (C-100) than eradicated (C-225) tumor. However, the TILs upregulated PD-1 and LAG-3 more potently and exhibited impaired effector functions in growing tumor compared to their counterparts in eradicated tumor. C-225 TME is highly enriched with myeloid cells, especially polymorphonuclear (PMN) myeloid-derived suppressor cells (MDSC), whereas the percentage of M-MDSC and tumor-associated macrophages (TAMs) was much higher in C-100 TME, especially M2-TAMs (CD206^+^). The complete eradication of C-225 depended on CD8 T cells and elicited anti-tumor memory responses upon secondary tumor challenge. We employed DNA sequencing to identify differences in the T cell receptor of peripheral blood lymphocytes pre- and post-secondary tumor challenge. Lastly, C-225 and C-100 tumor lines harbored different somatic mutations. Overall, we uncovered differential immune TME that may underlie the divergent outcomes of anti-tumor immunity by establishing two SCC tumor lines, both of which express HPV16 E6 and E7 antigens. Our experimental models may provide a platform for pinpointing tumor-intrinsic versus host-intrinsic differences in orchestrating an immunosuppressive TME in HNSCCs and for identifying new targets that render tumor cells vulnerable to immune attack.

## Introduction

Head and neck cancers (HNC) arise from the mucosal surfaces of the upper aerodigestive tract, and present as one of commonest types of cancer in the US and worldwide (1). Head and neck squamous cell carcinomas (HNSCCs) constitute 90% of all HNC (1, 2) and exhibit a high morbidity and mortality rate with a 5-year survival rate of ~50% (3). HNSCCs frequently associate with mutagens (e.g., tobacco or alcohol) or human papilloma virus (HPV) (4–6), thereby categorized as HPV^−^ or HPV^+^ HNSCCs. Oncogenic HPV subtypes (16 and 18) play a key role in the etiology of a subset of HNSCC, particularly those arising in the oropharynx (7–9). The incidence of HPV^+^ oropharyngeal HNSCC is rapidly rising (10). In the US, two-thirds of HNSCC patients with oropharynx cancer have HPV^+^ tumors (11). HPV^+^ HNSCC generally has a more favorable prognosis than HPV^−^ HNSCC (7, 11). Nevertheless, given the rapid rising of HPV^+^ HNSCC cases, it is important to develop new therapeutic strategies to treat HPV^+^ HNSCCs more effectively. HPV infection has been strongly associated with HNSCC development. A cohort of patients is unable to clear the HPV infection, which may lead to disease persistence, chronic inflammation and carcinogenesis (12). However, how HPV leads to HNSCC development in the context of adaptive immunity and the role of HPV antigen in mediating anti-tumor immunity remains incompletely understood.

The outcome of anti-tumor immunity can be highly heterogeneous with some hosts capable of eradicating their tumors while others succumbing to tumor progression. Despite extensive prior studies, the mechanisms underlying the heterogeneity of anti-tumor immune responses remain elusive. A deeper understanding of such mechanisms may have a positive impact on developing more effective personalized cancer immunotherapy. Human HNSCC samples exhibit a wide range of mutational burden and infiltration of immune cells including tumor-infiltrating myeloid cells in the tumor microenvironment (TME) (13–16). In general, HNSCC samples present with an immunosuppressive TME manifested with a high level of tumor-infiltrating myeloid cells such as tumor-associated macrophage (TAMs) and myeloid-derived suppressor cells (MDSCs) (16). It has been established that the two subsets of MDSCs, namely, M-MDSC (monocytic-MDSC) and PMN-MDSC (polymorphonuclear-MDSC), may play a pivotal role in immune suppression during tumorigenesis (17). Our prior studies also identified the infiltration of M-MDSC and PMN-MDSC in the TME of various mouse models of HNSCCs including KPPA tumor and A223 tumor (18–20).

It is well-known that HNSCC patients mount heterogeneous anti-tumor immune responses, evidenced by a highly variable level of T cell infiltration before treatment (21, 22). However, it is very difficult to model such heterogeneity in human patients due to uncontrollable variables including completely different genetic backgrounds, distinct immune systems, and vastly different tumors. Thus, a well-controlled mouse model may facilitate the investigation of mechanistic differences in anti-tumor immune responses in different individuals. To establish a mouse model that mimics HPV^+^ HNSCCs, we introduced HPV16 E6 and E7 into a mouse squamous cell carcinoma (SCC) line and generated two subclones, both of which express HPV16 E7 antigens (C-225 and C-100). Surprisingly, we found that C-225 and C-100 elicited completely different outcomes when transplanted into C57BL/6 recipient mice with C-225 eradicated and C-100 progressing aggressively. Complete eradication was dependent on CD8 T cells. We examined immune TME using flow cytometry and found that eradicated or growing tumors exhibited differential immune profiles that may influence the outcome of anti-tumor immunity. Our experimental models may provide a platform for elucidating tumor-intrinsic vs. host-intrinsic differences in setting up an immunosuppressive TME in HNSCCs and for discovering new targets that render tumor cells vulnerable to immune attack.

## Materials and methods

### Transfection and PCR

The plasmids containing the E6/E7 cDNA (pB-actin E6 E7, Catalog No. 13712, Addgene, USA) were transfected into the parental (A1419) cell line using the Amaxa™ 4D-Nucleofector™ Protocol for Normal Human Epidermal Keratinocytes (NHEK) (P3 Primary Cell 4D-Nucleofector™ X Kit, Program DS-138). A GFP-containing plasmid was co-transfected according to the manufacturer’s protocol. The GFP-positive cells were sorted using a cell sorter (Beckman Coulter MoFlo Astrios High Speed Sorter) into 96 well plates (single cell per well) and cultured to establish single clones of stable cell lines. To confirm the presence of E7 cDNA, genomic DNA isolated from the subclones was screened using PCR with the forward primer 5’-GAACCGGACAGAGCCCATTA-3’ and reverse primer 5’-TCTGAGAACAGATGGGGCAC-3’. The resultant PCR products were run on agarose gel and visualized using the G: Box Chemi-XX6 platform (Syngene, Frederick, MD). E7 positive clones were identified.

MEER, C-225 and C-100 tumor cell lines were used for the detection of E6 transcript. Total RNA was purified with Trizol reagent (Catalog15596026, Invitrogen). For each cDNA synthesis reaction, total template RNA (1μg) was used according to manufacturer’s instructions using Revert Aid First Strand cDNA Synthesis Kit (K1622, Thermo Scientific) followed by real-time PCR. Briefly, the reaction mixture contained 4μL of diluted cDNA (1:20) sample, 0.2μM Primers and 5μl of SYBR green mix. PCRs were performed in Light Cycler 480II (Roche, Basel, Switzerland). The primers and PCR reaction condition were listed in **Supplementary Table 3**. For quantification, percentage of E6 transcript/beta-actin was calculated based on the equation 100 × 2^Ct(beta-actin)-Ct (E6)^ and presented. Negative control was also included with each set of reactions, which contains all PCR reagents other than cDNA.

### Western blot and cell culture

Cells were harvested and lysed using a lysis buffer containing Tris-base (50 mM, pH 7.5), EDTA (1 mM), NaCl (150 mM), Sodium orthovanadate (2 mM), Sodium Fluoride (4 mM), Triton-X100 (1%), SDS (0.1%), and Sodium deoxycholate (0.5%). The lysate was incubated on ice for 30 min, and centrifuged at 12,000 rpm at 4°C for 10 min. The supernatant was collected, and protein concentration was quantified using a Pierce BCA protein assay kit (Catalog No. 23228, Thermo Scientific). The cellular protein (120μg) was loaded onto 12% gel and separated by SDS-PAGE (Bio-Rad, Hercules, CA). The separated proteins on SDS-PAGE were transferred onto nitrocellulose membranes (1620112, Bio-Rad). The membranes were blocked with 6% dried skimmed milk and subsequently probed with specific primary antibodies (anti-HPV16 E7 or anti-β-actin) followed by HRP-conjugated secondary antibodies (**Supplementary Table 1**). Protein bands were visualized using ECL Plus (Cytiva) on the G:Box Chemi-XX6 platform (Syngene, Frederick, MD).

Tumor cells were cultured in DMEM medium (Gibco, Thermo Fisher Scientific, USA) supplemented with 10% heat-inactivated FBS (fetal bovine serum) (Biowest, USA), antibiotic-antimycotic 100× (Gibco, Thermo Fisher Scientific, USA), and HEPES (Corning, USA) at 37°C CO_2_ incubator (5%). Cells were dissociated with trypsin (0.05%) (Gibco, Thermo Fisher Scientific, USA) and used for various experiments.

### *In vivo* animal studies, tumor injection, and histology

Wild-type (WT) C57BL/6 (B6) female mice were purchased from Charles River (6–8 weeks old). CD8-KO mice were purchased from Jackson Laboratory (JAX stock #002665) and genotyping PCR was performed as described on the JAX website (https://www.jax.org/Protocol?stockNumber=002665&protocolID=28916). All mice were maintained under specific pathogen-free conditions in the UPMC Hillman Cancer Center Animal Facility (Pittsburgh, PA). Animal work was approved by the Institutional Animal Care and Use Committee (IACUC) of University of Pittsburgh.

C-225 and C-100 tumor cells were cultured and trypsinized as described above. Cells were resuspended in sterile PBS and Matrigel (Corning, US) in a 1:1 ratio (50% PBS: 50% Matrigel). Subsequently, 2×10^6^ tumor cells (in a final volume of 100μL) were injected subcutaneously into the flank regions of each mouse. Tumor growth was monitored by measuring tumor volume (TV) with calipers and TV was calculated with the formula (length×width^2^)/2. C-225 tumors were harvested on day 7 when TV reached the maximal size, and C-100 tumors were collected on day 7 or day 30 after tumor inoculation. For histology analysis, tumors were fixed in 10% neutral-buffered formalin and submitted to Pitt Biospecimen Core/TARPS (University of Pittsburgh) for H&E staining. H&E slides were scanned, and images were captured using Keyence Digital Microscope (BZ-X810) (Magnification 20×).

### Flow cytometry

Single-cell suspensions were prepared from spleens or tumors harvested in culture medium from tumor-bearing mice as described previously (18). Briefly, tumors were finely cut and digested using Liberase DL (50μg/mL) (Roche, USA) for 30 mins at 37°C. The digested tumors or processed spleens were filtered through cell strainers, 70μm or 40μm, respectively. The red blood cells (RBC) in the single-cell suspensions were lysed using ACK lysis buffer (Quality Biological, USA), and neutralized with medium. Cells were washed and centrifuged at 1500 rpm for 5 mins at 4°C. The single-cell suspension was either stained for surface markers (see Ab List in **Supplementary Table 1**) or stimulated for intracellular cytokine staining (ICS). For ICS stimulation, the single-cell suspension of spleens or tumors was stimulated with ionomycin (650nM) and phorbol 12-myristate 13-acetate (PMA) (40nM) in the presence of Brefeldin A solution (1×) (BFA) (Catalog No. 347688, BD Biosciences, San Jose, CA, USA) in DMEM culture medium supplemented with β-mercaptoethanol (100µM) for 4 hrs at 37°C. Cells were first stained for LIVE/DEAD Fixable Aqua Dead Cell Stain (Invitrogen, Waltham, MA, USA) and blocked with TruStain FcX CD16/32 (BioLegend, San Diego, CA, USA) according to manufacturer’s instructions. Flow antibodies were employed at the concentrations according to the manufacturer’s recommendations. For ICS, Cytofix/CytoPerm buffer kit (BD Biosciences) was used to fix and permeabilize cells according to manufacturer’s instructions. Data were acquired on BD Fortessa and analyzed with FlowJo™ software (FLOWJO, Oregon, USA).

### Cell proliferation assay

The proliferation of C-225 and C-100 tumor cells was examined using CellTrace™ Violet Cell Proliferation Kit (Cat. No., C34557 Thermo Fischer Scientific) according to the manufacturer’s protocol. Cells were trypsinized and washed with PBS. Subsequently, 1×10^6^ cells were resuspended in 5µM CellTrace Violet in PBS, incubated in the dark at room temperature (RT) for 20 mins, neutralized by adding culture medium, and followed by 5 mins incubation at RT in the dark. Labeled cells were washed with medium and 4×10^4^ cells were seeded onto the culture plates (12 well) in triplicates. Cultured cells were harvested on day 0, day 1, day 2, and day 3, stained for live/dead cells using LIVE/DEAD™ Fixable Green Dead Cell Stain Kit (Cat. No. L23101, Invitrogen) and stored at 4°C in 1% paraformaldehyde until flow cytometry analysis. All samples collected on specified days were run on BD Fortessa on day 3 of cell culture. Data were analyzed using FlowJo™ software (FLOWJO, Oregon, USA).

### Whole exome sequencing and TCRβ sequencing

The genomic DNA samples were isolated from tumor cell lines including parental A1419, C-225 & C-100, and the DNA purity and concentration were determined by NanoDrop™ One^C^ (Thermo Fisher Scientific). DNA samples were submitted to Innomics for WES. Library prep was sequenced on DNBseq platform, and the read length was pair-end 150bp. Sequencing reads were analyzed as described previously (19). Raw reads underwent standard preprocessing steps: BWA alignment, sorting, marking duplicates and base quality score recalibration by using GATK (version 4.2.2.0). Two variant-calling pipelines, GATK Mutect2 function and BCFtools mpileup function were applied to identify tumor-specific variants using parental A1419 as normal control and C-100 or C-225 as tumor, respectively. Unique variants per cell line were annotated for SNPs and amino acid (protein) changes by tool SnpEff and SnpSift (23). For the first pipeline (GATK), further filtering was performed with FilterMutectCalls function after annotation and only passed variants were included. For the second pipeline (BCFtools), passed variants were defined as tumor total count >= 10, tumor alternative count >= 4, tumor alternative rate >= 0.1 and normal alternative rate <= 0.05. Variants with high or moderate impact were included that may alter protein functions or effectiveness.

PBMCs were isolated from peripheral blood samples collected from C-225 tumor-bearing mice (n=7) on day 102 (pre-PBMC) and day 116 (post-PBMC) after the first tumor inoculation. Each mouse has 2 samples sequenced including pre- and post-PBMC. In total, 14 samples were sequenced for 7 tumor-bearing mice. RBC was lysed as described above, and genomic DNA was purified and used for TCRβ sequencing with ImmunoSEQ platform by Adaptive Biotechnologies. ImmunoSEQ Analyzer was employed to retrieve, process, and track the TCRβ sequencing data. The data was further analyzed using R version 4.3.0 as described previously (24). Clonal proportion and clonotype tracking were analyzed with Immunarch (1.0.0) in R version 4.2.0.

## Results

### Generation of two HPV-antigen expressing SCC subclones that elicit opposite outcomes when transplanted *in vivo*

**Figure 1:**
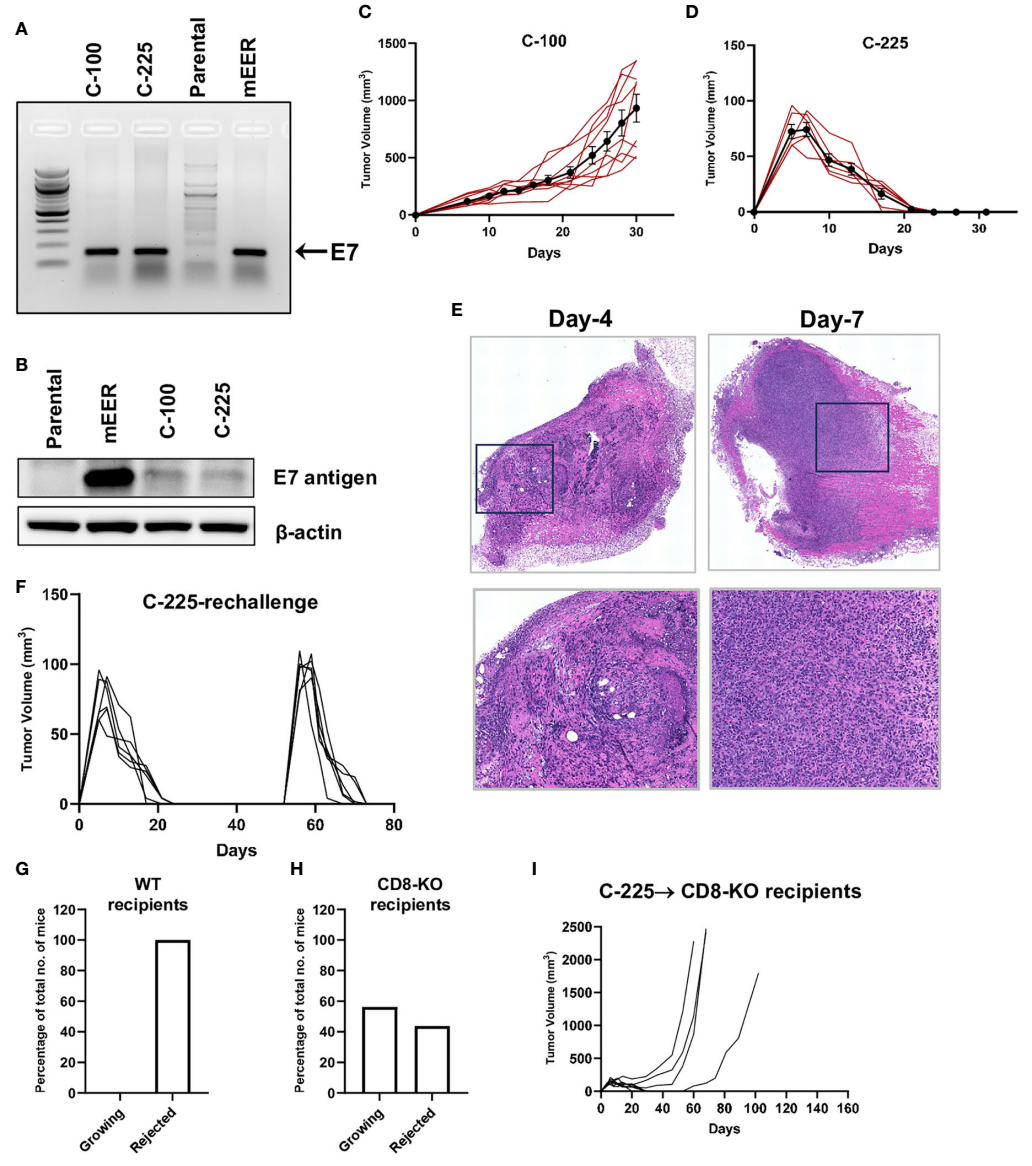
Generation of two SCC subclones eliciting opposite outcomes when transplanted *in vivo*. **(A)** Detection of E7 DNA. Representative gel image showing PCR products of E7 amplicons in C-100, C-225, and mEER (positive control) but not in parental A1419 tumor cells (negative control). **(B)** Detection of E7 protein. E7 protein expression detected by western blotting in mEER (positive control), C-100, and C-225 but not in parental A1419 tumor cells (negative control). **(C, D)** Tumor growth curve of C-100 **(C)** or C-225 **(D)** in WT B6 recipient mice. Tumor cells (2×10^6^) were inoculated at the flank region of WT B6 mice (n=9 for C-100; n=6 for C-225). **(E)** H&E analysis of C-225 tumors. Tumor samples were collected on day 4 and day 7 after tumor inoculation. Top panel: scan of the entire slides; Bottom panel: enlarged images of the selected region (black square in top panel). Magnification: 20×. **(F)** Tumor growth curve of first and secondary challenge with C-225 tumor cells. WT B6 mice (n=6) that rejected the first tumor challenged were re-challenged again on day 52 after the first tumor inoculation. **(G, H)** Percentage of recipient mice eradicating or succumbing to C-225 tumors. The percentage of WT B6 (n=13) **(H)** or CD8-KO (n=16) **(G)** mice that eradicated C-225 tumors or succumbed to tumor progression. **(I)** Tumor growth curve of CD8-KO recipient mice (n=8).

To establish a mouse model that can mimic HPV^+^ HNSCCs, we transfected HPV16 E6 and E7 cDNA into a SCC cell line (A1419) that was generated previously by 4-NQO induction (20). Upon transfection, we obtained two subclones, C-100 and C-225, which harbor E7 and E6 antigen confirmed by PCR (**Figure 1A**, data not shown), western blotting for E7 protein (**Figure 1B**) and real-time PCR for E6 transcript (**Supplementary Figure 1**). We used mEER cell line as a positive control, which was transformed by H-Ras and expressed HPV16 E7 antigen (25). Next, we transplanted C-225 and C-100 into wildtype (WT) B6 mice to test the tumor growth pattern *in vivo*. C-100 tumor grew out progressively in all of the recipient mice (**Figure 1C**). Surprisingly, we observed that the mice transplanted with C-225 tumor cells developed tumors initially; however, 100% of WT B6 recipient mice eliminated the tumor spontaneously without any intervention (**Figures 1D, G**). To confirm the initial tumor development upon C-225 injection, we performed H&E histological assessment of tumor samples collected at 4 or 7 days after tumor inoculation. We indeed observed the presence of tumor cells and C-225 tumors were characterized as moderately-to-poorly differentiated SCC (**Figure 1E**).

To test whether tumor eradication is mediated by adaptive immunity, we determined if mice that eradicated C-225 tumors develop immunological memory against C-225 tumors. We re-challenged a cohort of mice that had previously eradicated C-225 tumors with C-225 tumor cells and found that all of them eradicated tumors again with slightly faster kinetics (**Figure 1F**). To further test whether CD8 T cells play a critical role in mediating tumor eradication, we injected C-225 tumor cells into CD8^-/-^ mice and found that a fraction of these mice failed to eliminate tumors (**Figures 1H, I**), demonstrating that CD8 T cells were essential for complete tumor eradication. However, we also found that a portion of these mice were able to eliminate C-225 tumor (**Figure 1H**), and these data suggest that other immune cells such as CD4 T cells can also mediate tumor eradication. Overall, we conclude that C-225 tumor elicited complete eradication, which depends on CD8 T cells and induces an immunological memory response that quickly cleared a second tumor challenge.

### Characterization of CD4 and CD8 tumor-infiltrating lymphocytes in the immune TME of C-225 vs. C-100 tumors

**Figure 2:**
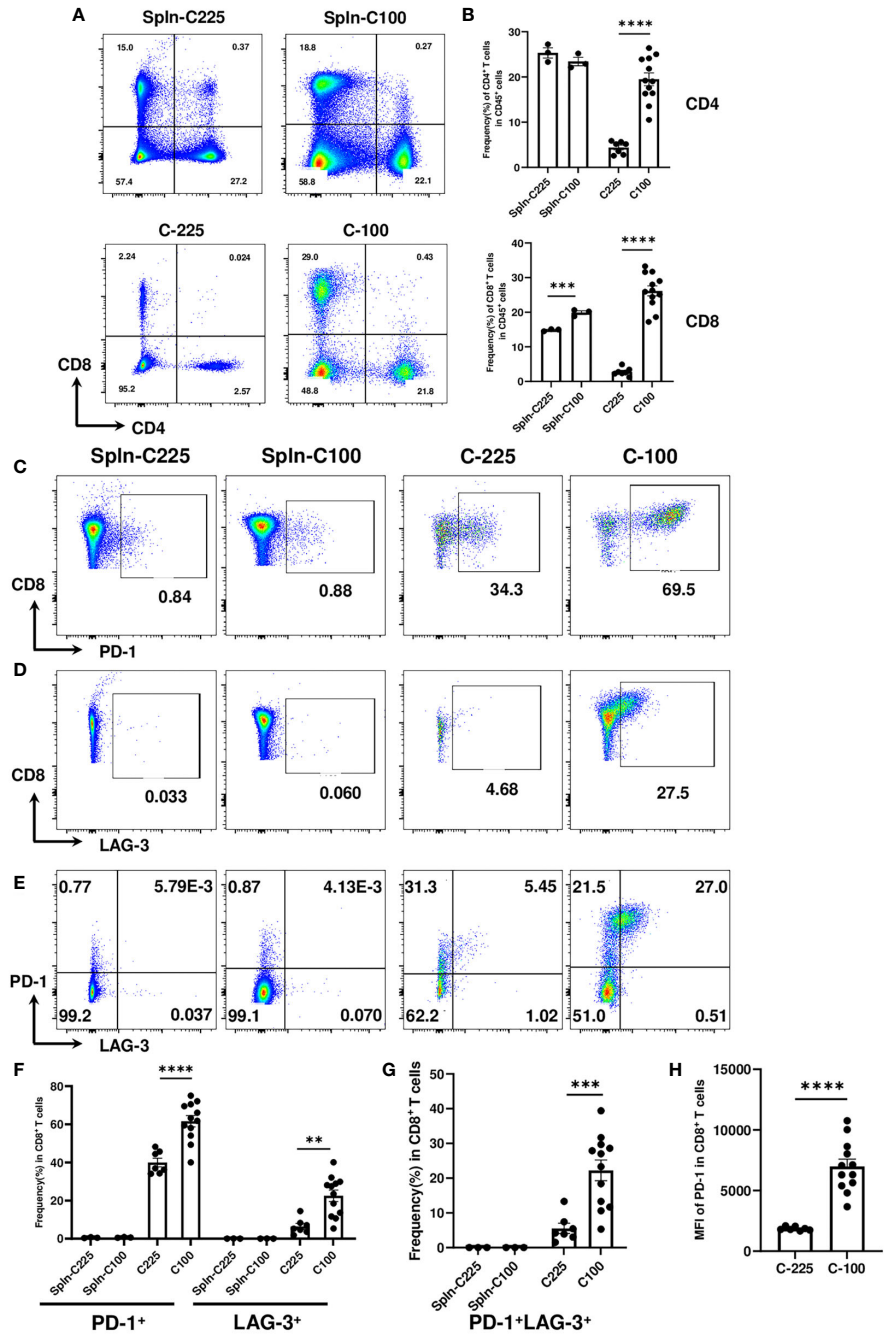
Characterization of CD4 and CD8 TILs in the TME of C-225 vs. C-100 tumors. Flow cytometry analysis was performed for spleen controls from tumor-bearing (TB) mice or CD4 and CD8 TILs from C-225 (n=7) or C-100 (n=12) tumors for all panels. Tumor and spleen samples were collected on day 7 or day 30 post-tumor injection, for C-225 or C-100, respectively. **(A)** Representative flow plots of CD4 and CD8 T cells in TB spleen (top panel) and tumors (bottom panel). **(B)** Quantification of the percentage of CD4^+^ or CD8^+^ T cells in CD45^+^ population of spleen controls, C-225 and C-100 tumors. **(C-H)** Differential expression of immune checkpoint molecules in CD8 T cells. Splenic CD8 T cells and CD8 TILs from C-100 and C-225 tumors were stained with anti-PD1 and anti-LAG-3 Abs. Representative flow plots are shown for CD8 vs. PD-1 **(C)**, CD8 vs. LAG-3 **(D)**, or PD-1 vs. LAG-3 (gated on CD8^+^ T cells) **(E)**. **(F, G)** Quantification of the percentage of CD8 T cells expressing different immune checkpoints in TB splenic control and tumors including PD-1 or LAG-3 **(F)** or both PD-1 and LAG-3 **(G)**. **(H)** A lower level of PD-1 expression in CD8 TILs of C-225 tumors shown by the MFI of PD-1. Statistical significance was calculated with an unpaired *t-*test; **P < 0.01, ***P < 0.001, **** <0.0001.

To better understand how C-100 (growing) and C-225 (eradicated) elicited opposite outcomes of anti-tumor immunity, we performed flow cytometry analysis to characterize CD4 and CD8 TILs in the TME. As controls, we analyzed the splenocytes collected from tumor-bearing (TB) mice with C-100 or C-225. The single-cell suspension of the TB spleen control, C-100 and C-225 tumors was stained with antibodies for CD4 and CD8 and gated on the CD45^+^ population (a marker for hematopoietic cells) to differentiate hematopoietic cells from other cell lineages (**Supplementary Figures 2A-D**). Surprisingly, we found that the percentage of CD4 and CD8 TILs within CD45^+^ population was significantly higher in C-100 than in C-225 tumors (**Figures 2A, B**).

To evaluate the expression level of immune checkpoint molecules, we performed flow cytometry analysis by comparing TB splenic control and CD8 TILs from C-225 or C-100 tumors. We found that TB splenic CD8 T cells expressed a minimal level of checkpoint molecules (PD-1 or LAG-3); in contrast, CD8 TILs upregulated PD-1 or LAG-3 (**Figures 2C, D, F**). Moreover, the percentage of PD-1^+^ or LAG-3^+^ CD8 TILs was significantly higher in C-100 than in C-225 tumors (**Figure 2F**). We also detected CD8 TILs co-expressing PD-1 and LAG-3 in the TME, the percentage of PD-1^+^LAG-3^+^ CD8 TILs was remarkably higher in C-100 than in C-225 tumors (**Figures 2E, G**). Furthermore, the MFI of PD-1 expression appeared to be much higher in the CD8 TILs of C-100 tumors than C-225 ones (**Figure 2H**). Taken together, our data suggest that CD8 TILs in C-100 tumors exhibited more exhausted phenotypes, consistent with tumor progression.

### Reduced effector functions of CD4 and CD8 TILs in C-100 tumors

**Figure 3:**
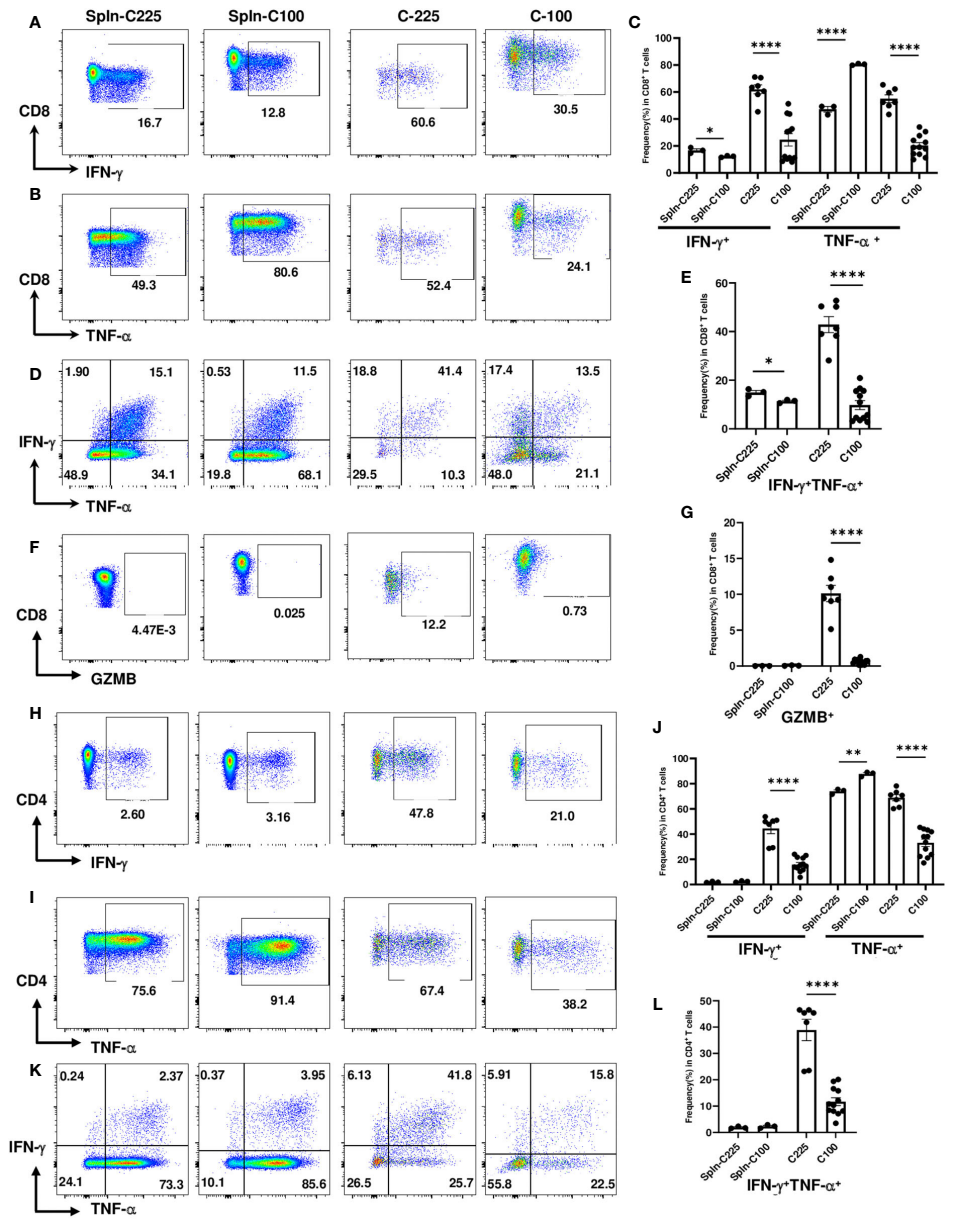
Reduced effector functions of CD4 and CD8 TILs in C-100 tumors. Flow cytometry analysis was performed as described in **Figure 2** (n=7 for C-225; n=12 for C-100). **(A-G)** Effector molecule expression in CD8 T cells. Representative flow plots for the expression levels of IFN-γ **(A)**, TNF-α **(B),** or IFN-γ^+^TNF-α^+^ **(D)** in CD8 T cells. **(C, E)** Quantification of the percentage of CD8 T cells expressing IFN-γ or TNF-α **(C)** or both IFN-γ^+^TNF-α^+^ **(E)** in TB splenic control or C-100 and C225 tumors. **(F, G)** Expression of GZMB in CD8 T cells. Representative flow plots of CD8 T cells expressing GZMB **(F)**. Quantification of the percentage of CD8 T cells expressing GZMB **(G)** in different groups. **(H-L)** Effector molecule expression in CD4 T cells. Representative flow plots for the expression levels of IFN-γ **(H)**, TNF-α **(I),** or IFN-γ^+^TNF-α^+^ **(K)** in CD4 T cells. **(J, L)** Quantification of the percentage of CD4 T cells expressing IFN-γ or TNF-α **(J)** or both IFN-γ^+^TNF-α^+^ **(L)** in different groups. Statistical significance was calculated with an unpaired *t-*test; *P < 0.05 **P < 0.01.

IFN-γ and TNF-α are effector cytokines commonly examined for T cell functions, especially for their polyfunctionality. Polyfunctional T cells are effector T cells that can produce different cytokines, retain cytotoxic potential and may be more effective in suppressing tumor growth (26, 27). The loss of double producers (IFN-γ^+^TNF-α^+^) is an indicator of CD8 T cell dysfunction (28). To investigate the functional changes in CD8 TILs, we performed intracellular cytokine staining to detect the percentage of IFN-γ^+^, TNF-α^+^ or IFN-γ^+^TNF-α^+^ CD8 T cells from TB splenic control or C-100 vs. C-225 tumors (**Figures 3A-E**). We found that CD8 TILs from C-100 tumors exhibited a lower level of effector functions compared to those from C-225 tumors, evidenced by a lower percentage of IFN-γ^+^ or TNF-α^+^ CD8 TILs (**Figures 3A-C**). Importantly, the percentage of double producers (IFN-γ^+^TNF-α^+^) was substantially lower in CD8 TILs from C-100 tumors than those from C-225 tumors (**Figures 3D, E**). Most splenic CD8 T cells are naïve CD8 T cells, and naïve CD8 T cells tend to produce a high level of TNF-α upon stimulation (29). Consistently, we found that the percentage of TNF-α^+^ CD8 T cells was high in the TB splenic CD8 T cells (**Figures 3B, C**). Granzyme B (GZMB) is another effector molecule produced by activated CD8 T cells. We found that the percentage of GZMB^+^ CD8 TILs was significantly higher in C-225 than C-100 tumors (**Figures 3F, G**). We concluded that the effector functions of CD8 TILs were significantly reduced in C-100 tumors compared to C-225 ones.

Given that C-225 eradication also occurred in CD8^-/-^ mice suggesting a contribution from immune cells other than CD8 T cells, we examined the effector functions of CD4 T cells from TB splenic control, C-225 or C-100 tumors. Consistent with data from CD8 TILs, we found that CD4 TILs in C-100 tumors also exhibited reduced effector functions compared to their counterparts in C-225 tumors, as shown by a lower percentage of IFN-γ^+^ or TNF-α^+^ CD4 TILs in C-100 (**Figures 3H-J**). More importantly, the percentage of double producers (IFN-γ^+^TNF-α^+^) in CD4 TILs was also substantially lower in C-100 than C-225 tumors (**Figures 3K, L**). Of note, we detected a higher percentage of GZMB^+^ CD4 TILs in C-225 than C-100 tumors (**Supplementary Figure 3A**). Overall, our results are consistent with the observation that CD8 and CD4 TILs in C-100 tumors were more exhausted with impaired effector functions compared to their counterparts in C-225 tumors.

### The drastic expansion of PMN-MDSCs in the TME of C-225 tumors

**Figure 4:**
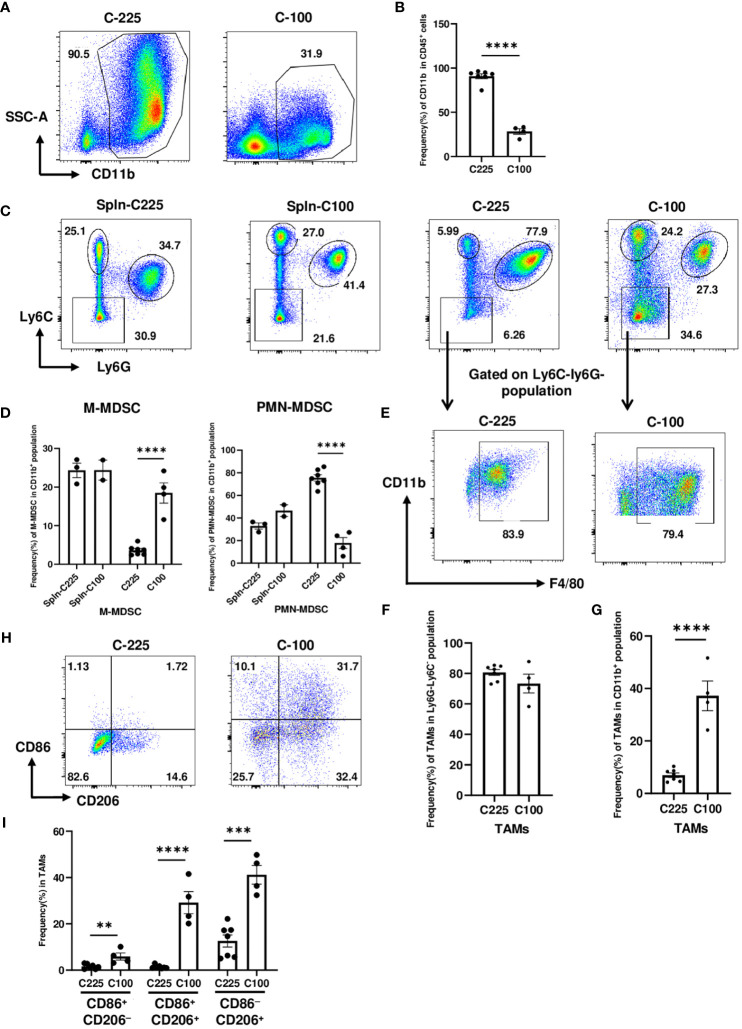
The drastic expansion of PMN-MDSCs in the TME of C-225 tumors. Flow cytometry analysis was performed for TB splenic control or tumor samples from tumor-bearing mice with C-225 (n=7) or C-100 (n=4) tumors, respectively, for all panels. **(A, B)** Representative flow plots for CD11b^+^ population within CD45^+^ population in C-225 or C-100 tumors **(A)**. Quantification of the percentage of CD11b^+^ population within CD45^+^ population **(B)**. **(C, D)** Analysis of different subsets of MDSCs (gated on CD11b^+^ population). Representative flow plots **(C)** for different subsets: M-MDSC (Ly6C^high^Ly6G^−^), PMN-MDSC (Ly6C^low^Ly6G^+^), and Ly6C^−^Ly6G^−^. Quantification of the percentage of M-MDSC or PMN-MDSC within CD11b^+^ population in indicated groups **(D)**. **(E)** Representative flow plots for TAM population gated on Ly6C^−^Ly6G^−^ population in panel C and displayed for CD11b vs. F4/80. **(F, G)** Quantification of the percentage of TAMs (CD11b^+^F4/80^+^Ly6C^−^Ly6G^−^) within Ly6C^−^Ly6G^−^ population **(F)** or within total CD11b^+^ population **(G)**. **(H)** Representative flow plots for TAMs (CD11b^+^Ly6C^−^Ly6G^−^F4/80^+^) expressing CD86 and/or CD206 in C-225 or C-100 tumors. **(I)** Quantification of the percentages of M1 (CD86^+^CD206^−^) and M2 (CD86^−^CD206^+^) TAMs. Statistical significance was calculated with an unpaired *t-*test; **P < 0.01, ***P < 0.001, **** <0.0001.

Apart from T cells, we also examined the tumor-infiltrating myeloid cells in C-225 vs. C-100 tumors. Our flow analysis showed that the percentage of CD11b^+^ population within CD45^+^ hematopoietic cells was significantly higher in C-225 than C-100 tumors (**Figures 4A, B**). Prior studies suggest that the two subsets of MDSCs, M-MDSC and PMN-MDSC, play a key role in immune suppression during tumorigenesis (17). By gating on the CD11b^+^ population with gating strategies established previously (**Supplementary Figure 2**) (17, 30), we assessed the percentage of M-MDSC (CD11b^+^Ly6G^−^Ly6C^high^) vs. PMN-MDSC (CD11b^+^Ly6G^+^Ly6C^low^) and the percentage of CD11b^+^Ly6C^−^Ly6G^−^ population in both C-225 and C-100 tumors and in TB splenic controls (**Figure 4C**). We found that the percentage of M-MDSC population was remarkably higher in C-100 than C-225 tumors; in contrast, the percentage of PMN-MDSC was significantly higher in C-225 than in C-100 tumors (**Figure 4D**). Thus, C-225 tumors exhibit a preferential increase of PMN-MDSC, whereas C-100 tumors are infiltrated with more M-MDSC.

By gating on Ly6C^−^Ly6G^−^ population, we found that most of this population was composed of TAMs (CD11b^+^F4/80^+^Ly6C^−^Ly6G^−^) (**Figure 4E**). The percentage of TAMs in Ly6C^−^Ly6G^−^ population did not differ significantly between C-225 and C-100 tumors (**Figure 4F**); however, the percentage of TAMs in CD11b^+^ population was significantly higher in C-100 than C-225 tumors (**Figure 4G**). TAMs can be further classified into subsets based on surface markers such as M1-TAMs (CD86^+^CD206^−^), which may mediate proinflammatory and anti-tumor responses, and M2-TAMs (CD206^+^), which are thought to be immunosuppressive and promote tumor growth [26, 28]. Our results showed that, within the TAM population (CD11b^+^F4/80^+^ Ly6C^−^Ly6G^−^), the percentage of CD86^+^CD206^−^, CD86^+^CD206^+^ or CD206^+^CD86^−^ population was significantly higher in C-100 tumors, while TAMs in C-225 tumors did not express a high level of CD86 or CD206 (**Figures 4H, I**). These data demonstrate that TAMs in C-100 tumors exhibit phenotypes consistent with immunosuppression.

### Dynamic changes in the TILs of C-100 tumors harvested at an earlier time point

The dynamic changes in the frequency and function of TILs make it difficult to choose the optimal time point for comparing the growing C-100 and regressing C-225. As the tumor size increases, the changes in the myeloid compartment may further complicate our ability to compare these two tumors effectively. Therefore, we initially chose to compare these two tumors by focusing on the outcome of tumor growth, basically, with the former showing a full-blown tumor progression (day30) and the latter being suppressed (day7). This scenario of comparison may allow us to reveal the differences between a completely failed anti-tumor immune response vs. a successful one. Nevertheless, harvesting C-100 tumor at an earlier time point may provide more insights into the signs of early exhaustion in TME.

Therefore, we performed the experiments by inoculating C-100 tumors into WT B6 mice and harvested the tumors for flow analysis at day 7 post tumor injection and compared the immune phenotypes of these C-100 tumors (C-100-day-7) with the C-225 tumors harvested at day 7 (C-225-day-7) and the C-100 tumors harvested at day 30 (C-100-day-30). The tumor volume was similar between C-100-day-7 and C-225-day-7 (**Supplementary Figure 4**). There was a significantly higher level of CD4 and CD8 TILs in C-100-day-30 than either C-100-day-7 or C-225-day-7 (**Supplementary Figures 5A, B**). The percentage of PD-1^+^ CD8 TILs was significantly higher in C-100-day-30 than in either C-100-day-7 or C-225-day-7 (**Supplementary Figure 5C**). In contrast, the percentage of LAG-3^+^ or PD-1^+^LAG-3^+^ CD8 TILs was significantly higher in both C-100-day-7 and C-100-day-30 than C-225-day-7 (**Supplementary Figures 5C, D**). These data suggest that LAG-3 expression may serve as an early sign of CD8 TIL exhaustion in the TME of growing tumor.

We next compared the cytokine production of IFN-γ, TNFα, or both in CD4 and CD8 TILs. For CD8 TILs, the percentage of IFN-γ^+^, TNF-α^+^ or IFN-γ^+^TNF-α^+^ CD8 TILs did not differ between C-100-day-7 or C-225-day-7, while both groups were significantly higher than C-100-day-30 (**Supplementary Figures 6A, B**). In contrast, the percentage of GZMB^+^ CD8 TILs was significantly higher in C-225-day-7 than in both C-100-day-7 and C-100-day-30 (**Supplementary Figure 6C**), suggesting that the lack of GZMB expression may be an early sign of CD8 TIL exhaustion. For CD4 TILs, the percentage of IFN-γ^+^, TNF-α^+^ or IFN-γ^+^TNF-α^+^ CD4 TILs was significantly higher in C-225-day-7 than in both C-100-day-7 and C-100-day-30 (**Supplementary Figures 6D, E**). Similarly, the percentage of GZMB^+^ CD4 TILs was significantly higher in C-225-day-7 than in both C-100-day-7 and C-100-day-30 (**Supplementary Figure 6F**). These data suggest that the decline of cytokine production in CD4 TILs at day 7 may be an early sign of T cell exhaustion in the TME of growing tumors.

We found that the percentage of CD11b^+^ population within CD45^+^ hematopoietic cells decreased from C-225-day-7 to C-100-day-7, then to an even greater extent in C-100-day-30 (**Supplementary Figure 7A**). Similarly, the percentage of PMN-MDSC within CD11b^+^ population exhibited a gradually decreasing pattern from C-225-day-7 to C-100-day-7, then to C-100-day-30 (**Supplementary Figure 7C**). However, the percentage of M-MDSC within CD11b^+^ population was significantly increased in both C-100-day-7 and C-100-day-30 compared with C-225-day-7 (**Supplementary Figure 7B**), suggesting that the early rise of M-MDSC at day 7 may be a sign of developing immunosuppressive TME. Also, the percentage of TAMs within CD11b^+^ population was significantly higher in C-100-day-30 than either C-225-day-7 or C-100-day-7 (**Supplementary Figure 7D**), indicating its correlation with the immunosuppressive TME of a full-blown tumor. In contrast, the percentage of TAMs within Ly6C^−^Ly6G^−^ population did not correlate with tumor growth pattern (**Supplementary Figure 7E**). TAMs can be further divided into subsets based on surface markers. Within the TAM population (CD11b^+^F4/80^+^Ly6C^−^Ly6G^−^), the percentage of CD86^+^CD206^−^, CD86^+^CD206^+^ or CD206^+^CD86^−^ population varied substantially among three groups (**Supplementary Figure 7F**). Notably, the percentage of CD86^+^CD206^+^ population was increased in both C-100-day-7 and C-100-day-30 compared to C-225-day-7 (**Supplementary Figure 7F**), indicating that the early rise of this population may serve as a marker of developing immunosuppressive TME. Taken together, this illustrates C-225-day-7 exhibit a preferential increase of PMN-MDSC, whereas C-100-day-7 and C-100-day-30 are enriched with M-MDSC and distinct subsets of TAMs, whose early rise may serve as markers of developing immunosuppressive TME.

### T cell receptor dynamic changes upon secondary tumor challenge of C-225

**Figure 5:**
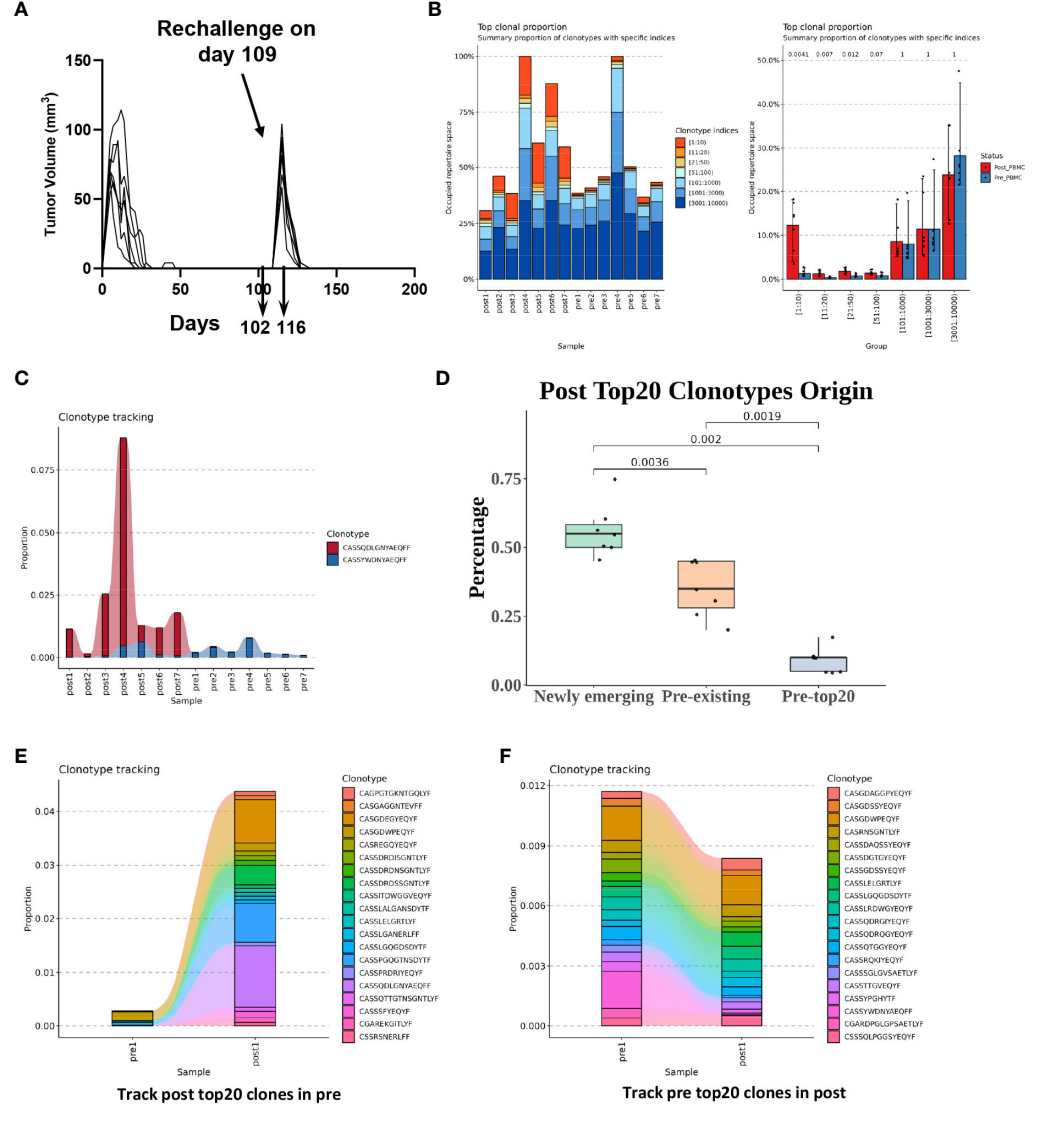
T cell receptor (TCR) dynamic changes upon secondary challenge of C-225 tumor cells. **(A)** Schematic of tumor challenge and sample collection. WT B6 mice (n=7) that eradicated C-225 tumors were challenged with C-225 tumor on day 109 after the first tumor inoculation. Blood samples were collected 7 days before (day 102, pre-challenge, n=7) and 7 days after rechallenging (day 116, post-challenge, n=7). **(B)** Greater TCRβ clonal expansion in post-challenge (post1-post7) samples than pre-challenge (pre1-pre7) ones. Left: individual bar graph representing the occupied repertoire space for each sample sequenced with the proportion of each group’s TCRβ clonotypes shown color-coded according to clonotype indices such as 1:10, 11:20 or 21:50. Right: TCR clonotypes were sorted into different groups according to specific indices such as 1:10 or 11:20. The proportion of the clonotypes in each group was averaged for 7 pre- or 7 post-challenge samples (Pre-PBMC vs. Post-PBMC). P values are listed on top of each bar graph. Statistical difference was calculated with Wilcoxon rank sum test. **(C)** Clonotype tracking for the prevalent TCRβ clonotypes detected in all 7 pre- or 7 post-challenge samples. The proportion of a given TCRβ clonotype within each sample is shown along the y axis while sample ID is shown along x axis. **(D)** Newly emerging TCR clonotypes dominate in the top-ranked 20 clones in post-challenge samples. The top-ranked 20 clones from 7 post-challenge samples (n=140) were separated into three groups based on their clonal frequency in pre-challenge samples: newly emerging (clonal frequency=0), pre-existing (clonal frequency>0 but not in pre-top 20) and Pre-top20 (ranked in top 20 clones in the corresponding pre-challenge sample). The percentage of clonotypes in each group is shown. **(E)** Clonotype tracking of post-challenge (post 1) top-ranked 20 clones in corresponding pre-challenge samples (pre1). **(F)** Clonotype tracking of pre-challenge (pre 1) top-ranked 20 clones in corresponding post-challenge sample (post 1).

TCRs are generated via a somatic DNA recombination process, termed V(D)J recombination (31, 32). TCRs of most conventional T cells consist of an alpha (α) chain and a beta (β) chain, encoded by *TRA* and *TRB*, respectively, and linked by disulfide bonds. TCRs can be grouped into different “clonotypes” which have unique TCRα or TCRβ chains containing distinct V(D)J gene segments and complementarity-determining region 3 (CDR3). CDR3 region encompasses the highly divergent junction of V(D)J recombination and plays a key role in antigen recognition. To determine the dynamic changes in TCRs upon secondary tumor challenge, we inoculated C-225 tumor cells into the recipients that had rejected the first tumor challenge at 109 days after the first tumor inoculation (**Figure 5A**). PBMC samples were collected 7 days before (day 102) and after (day 116) the second tumor inoculation (**Figure 5A**). CD3^+^ T cells were purified from PBMC, and genomic DNA was obtained and subjected to TCRβ CDR3 DNA sequencing using Adaptive Biotechnologies’ immunoSEQ platform, which allowed us to examine more productive TCRβ CDR3 sequences. In total, we sequenced 7 pre-challenge (pre1-pre7) and 7 post-challenge (post1-post7) samples by isolating PBMC T cells from 7 individual mice. The total numbers of sequenced templates and productive rearrangements were shown for all 14 samples (**Supplementary Table 2**).

We found that T cells from post-challenge PBMC samples (post1-post7) underwent clonal expansion to a much greater degree than pre-challenge ones (**Figure 5B**, left). In particular, the relative abundance of top-ranked 20 clones (1:10 and 11:20) was remarkably higher in post-challenge samples compared to pre-challenge ones (**Figure 5B**, right). Of note, we identified one TCRβ clonotype (CASSQDLGNYAEQFF) whose clonal proportion was very high in some of the post-challenge samples and was present in the top-ranked 20 clones from all post-challenge samples (post1-post7). We also identified one TCRβ clonotype (CASSYWDNYAEQFF) that was present in the top-ranked 20 clones from all pre-challenge samples (pre1-pre7) (**Figure 5C**).

To better delineate the origin of top-ranked 20 clones in post-challenge samples, we categorized the TCR clonotypes into three groups: newly emerging, pre-existing, and pre-top20 (**Figure 5D**). We found that the majority (~55%) of the top-ranked 20 clones in post-challenge samples were newly emerging, which means that they were not detected in pre-challenge samples (**Figure 5D**). About 35% of the top-ranked 20 clones were pre-existing, which means that they were detected in the pre-challenge samples. Only about 10% of the top-ranked 20 clones fell in the category of pre-top20, which means that they were detected in the top-ranked 20 clones from pre-challenge samples (**Figure 5D**). When we performed clone tracking analysis, we found that the top-ranked 20 clones from post-challenge samples were almost undetectable in pre-challenge samples except for very few clones (**Figure 5E**; **Supplementary Figure 8**). In contrast, we found that the clonal proportions of top-ranked 20 clones from pre-challenge samples were collectively reduced in post-challenge samples and did not show substantial differences between pre- and post-challenge samples (**Figure 5F**; **Supplementary Figure 8**). Taken together, we conclude that T cells in post-challenge samples underwent substantial clonal expansion, and most of the top-expanded clones (top-20 clones) were newly emerging or pre-existing at a very low frequency and did not overlap with the pre-existing top-20 clones in pre-challenge samples.

### Tumor-intrinsic differences in C-100 and C-225

**Figure 6:**
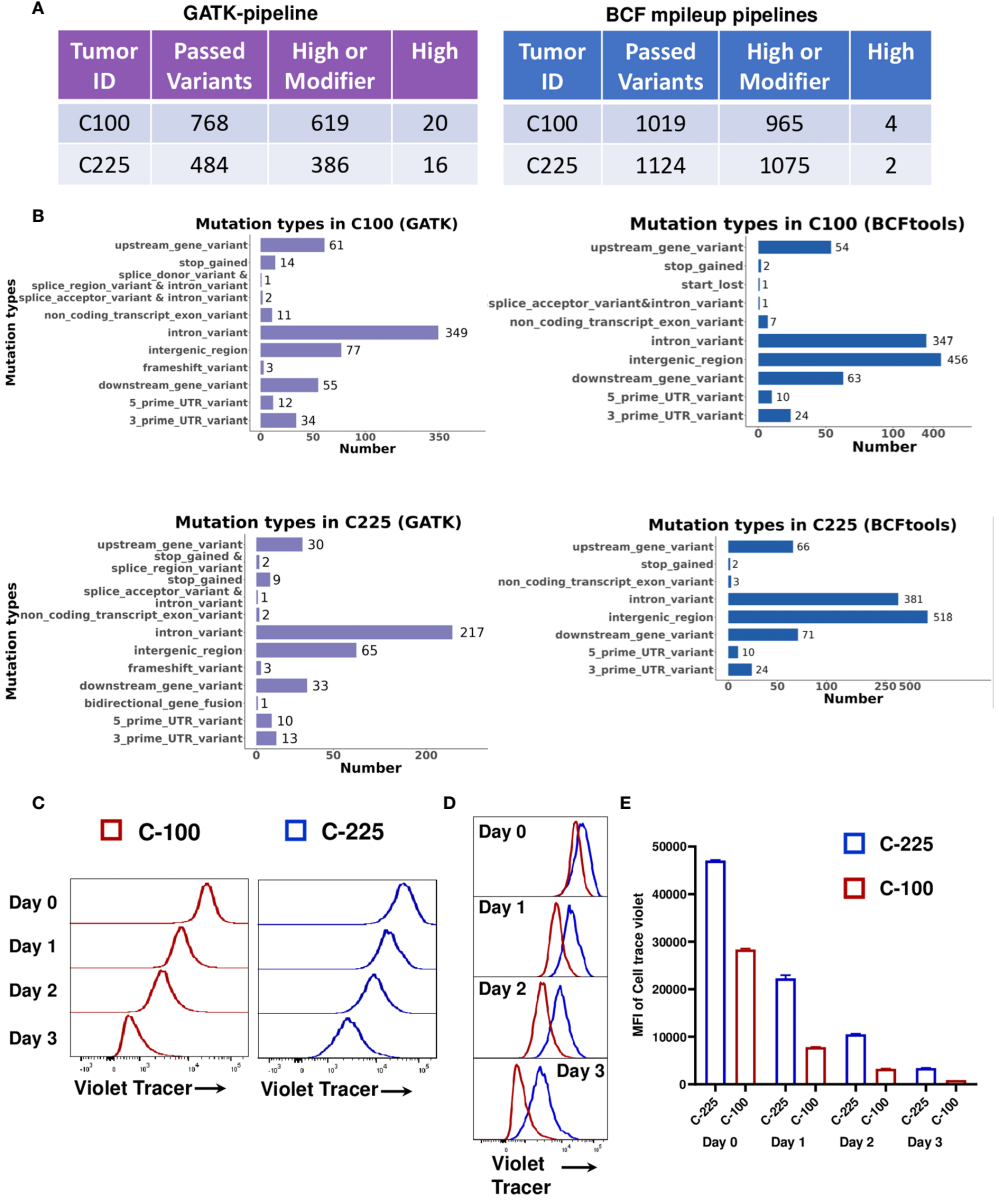
Tumor-intrinsic differences in C-100 and C-225 tumor cells. **(A)** WES analysis by GATK-pipeline (left) and BCFtools mpileup-pipeline (right) revealed different numbers of variants in C-100 or C-225 tumor cells compared to parental A1419 tumor cells. **(B)** Mutation types in C-100 or C-225 tumor cells. Number of mutations identified in each category in C-100 or C-225 tumor cells using either GATK or BCFtools mpileup pipeline. **(C-E)** Tumor cell proliferation assays. Tumor cells were labeled with CellTrace™ Violet and cultured for 1, 2, and 3 days. Cells harvested on specific days along with day 0 samples were analyzed by flow cytometry. Populations with less intensity of CellTrace™ Violet staining (C-100) indicate more cell divisions. **(C)** Representative histogram for the proliferation of C-100 (red) and C-225 (blue) tumor cells on indicated days. **(D)** Overlay of the histogram of C-225 vs. C-100 for comparison of cell proliferation on indicated days. C-100 tumor cells exhibited less intensity (more dilution of CellTrace™ Violet) indicating more proliferation. **(E)** Quantification of mean fluorescence intensity (MFI) of CellTrace™ Violet in C-225 (blue) and C-100 (red) tumor cells on indicated days. Statistical significance was calculated by student’s t test, p value for C-225 vs. C-100 on day 0 = 0.0002, day 1 < 0.0001, day 2 < 0.0001, and day 3 < 0.0001.

To determine if C-100 and C-225 harbor different genetic mutations, we performed WES of C-100, C-225 and parental SCC (A1419). Using A1419 as control, we identified genetic differences between C-100 and C-225 tumor lines in the WES data that were independently analyzed using two different pipelines (see details in Method). Both analyses showed that C-100 and C-225 tumors contained tumor-specific somatic mutations when compared to parental A1419 SCC line (**Figures 6A, B**). All of the identified somatic mutations were listed in **Supplementary Tables 4**, **5** (analysis I, GATK pipeline) and **Supplementary Tables 6**, **7** (analysis II, BCFtools/mileup pipeline). Thus, we suggest that C-100 and C-225 tumors harbor tumor-specific genetic mutations that may contribute to their differential TME. Next, we tested whether C-100 and C-225 tumor cells differ in their proliferative abilities. Our data showed that C-100 tumor cells proliferate faster than C-225 ones (**Figures 6C-E**), consistent with the *in vivo* aggressive tumor growth of C-100 (**Figure 1C**). Overall, we suggest that tumor-intrinsic differences may account for differential TME of C-100 vs. C-225 tumors *in vivo*.

## Discussion

We presented a model system to compare the HPV-antigen expressing SCC lines that elicited opposite outcomes of anti-tumor immunity, namely, tumor eradication vs. tumor progression, which associated with differential immune profiles in the TME. We made unexpected findings that the percentage of CD8 and CD4 TILs was much lower in eradicated (C-225) tumor than growing one (C-100); nevertheless, the TILs in C-225 exhibited a lower level of PD-1 and LAG-3 expression and demonstrated more robust effector functions than their counterparts in C-100. On one hand, C-225 eradication depended on CD8 T cells since not all of the CD8 KO recipient mice eradicated tumors. On the other hand, immune cells besides CD8 T cells, such as CD4 T cells, also contributed to tumor eradication since ~40% of CD8-KO recipient mice did eradicate tumor. In line with this idea, we observed that CD4 TILs displayed stronger effector functions in the TME of C-225, consistent with prior studies showing a role of CD4 T cells in anti-tumor immunity (33–36). While frequency of CD4 T cells was associated with better prognosis in HNSCC, the specific role of CD4 T cells in HNSCC anti-tumor immunity remain less well-understood (15, 37, 38). Future studies are warranted to explore the role of CD4 TILs in this model system. It is also puzzling that only a fraction of CD8-KO mice eradicated tumors, suggesting that differences in the immune system of the individual host may account for the variable responses (39–42).

Notably, the TME of C-225 tumors is completely dominated by myeloid cells, with ~90% of CD45^+^ population being CD11b^+^ (**Figures 4A, B**). Among the CD11b^+^ population, 80% of them was PMN-MDSC, whereas the percentage of M-MDSC and TAMs was much lower (~15%). In TAM population, the percentages of both CD86^+^ and CD206^+^ populations were much lower in C-225 TME. Taken together, our data suggest that PMN-MDSC may not be a major contributor to immunosuppression and may be able to mediate anti-tumor responses under certain circumstances, at least in our model system. This notion is contrary to the prevailing view that PMN-MDSC is an immunosuppressive population (16, 17). However, a recent study reports that PMN-MDSC isolated from tumor-bearing mice treated with ceralasertib, an ATR inhibitor, exhibited a significantly lower suppressive activity against CD8 T cells, and the reduced suppressive activity was associated with up-regulation of type I IFN signature in PMN-MDSC (43). Taken together, we suggest that the function of PMN-MDSC can be modulated by therapeutic agents that skew this population to be permissive of anti-tumor immunity. In line with prior studies (16, 44), our data support an immunosuppressive role of TAMs, especially so-called M2-TAMs (CD206^+^), during progression of SCC tumors. M2-TAMs express a higher level of CD206 and can carry out immunosuppressive functions by expressing arginase-1 (Arg-1), chemoattractant such as IL-10 and TGF-β, and chemokine CCL17 and CCL22 (45). Consistently, the TME of HNSCC largely consists of M2-TAMs, which could reduce effector T cell function (46). A greater percentage of TAMs in the TME associates with lymph node metastasis and advanced stage of HNSCCs (16, 47). We suggest that future targeting approaches should be geared toward reducing TAMs or M-MDSC but modulating PMN-MDSC for HNSCC immunotherapy.

Our analysis of dynamic changes in the TILs of C-100 tumors harvested at different time points identified a few early indicators of immunosuppressive TME. We found that LAG-3 expression may serve as an early sign of CD8 TIL exhaustion while a decline of cytokine production in CD4 TILs at day 7 may indicate CD4 TIL exhaustion. An early rise of M-MDSC and distinct subsets of TAMs (CD86^+^CD206^+^ population) at day 7 may serve as markers of developing immunosuppressive TME. Future studies are warranted to better characterize different subsets of immune cells in C-100 vs. C-225 tumors at day 7 with more granular approaches. These studies may elucidate underlying mechanisms that dictate the outcomes of anti-tumor immunity at an early time point.

We noticed that the results of WES appear to be different between the two pipelines employed for data analysis. The two pipelines, GATK and BCFtools, will yield different mutation calling and distributions because they employ different approaches to identify and call variants (48, 49). While a systematic comparison of the two pipelines is beyond the scope of our current study, there are notable differences between these two pipelines: GATK Haplotypecaller is a local reassembly of haplotypes, while BCFtools is a positional and pileup based variant caller. These two pipelines also use different ways to perform filtering steps which filter out the potentially false-positive variants (48, 49).

We employed DNA sequencing to identify differences in the TCR of peripheral blood lymphocytes pre- and post-secondary challenge of C-225 tumor. It is not surprising that TCRs in post-challenge samples underwent more clonal expansion; however, it is unexpected that the top-ranked 20 clones are mainly composed of newly emerging clones, and very few of them overlap with the top-ranked 20 clones detected in pre-challenge samples. Our data suggest that new TCRs are preferentially recruited to anti-tumor memory responses (recall), instead of pre-existing top-ranked TCR clones (primary memory). This finding is distinct from previous studies that clearly showed consistent selection of the same TCRαβ clonotypes following secondary virus infection by comparing the TCR clonotypes from primary memory vs. recall responses (50).

The role of HPV antigen in tumor eradication remains unclear. Both C-225 and C-100 expressed a low level of E7 antigen, while mEER expressed a high level of E7 protein and E6 transcript but failed to induce tumor eradication. Thus, we suggest that the mere presence of HPV antigens is not sufficient to mediate tumor eradication; however, it remains possible that HPV antigen contributes to tumor eradication of C-225 because C-225 expressed a higher level of E6 transcript than C-100 (**Supplementary Figure 1**). Lastly, C-225 and C-100 tumor cell lines harbored different somatic mutations compared to parental A1419, suggesting that tumor-intrinsic mechanisms may play a role in mediating tumor eradication by orchestrating a less immunosuppressive TME (19, 41, 51, 52). Further studies are needed to pinpoint the tumor-intrinsic factors that influence the differentiation or expansion of tumor-infiltrating myeloid cells, especially for PMN-MDSC. Our experimental models may provide a platform for identifying tumor-intrinsic vs. host-intrinsic differences in influencing the outcome of anti-tumor immunity in HNSCCs and for uncovering novel targets that may render tumor cells vulnerable to immune attack mediated by various populations including CD8, CD4 and PMN-MDSCs.

## Data availability statement

The WES data presented in the study are publicly available and deposited in NCBI Sequence Read Archive (SRA) (BioProject: PRJNA1124061). The TCR sequencing data are deposited in GEO database with GEO Submission (GSE271371).

## Ethics statement

The animal study was approved by the Institutional Animal Care and Use Committee of University of Pittsburgh (Pittsburgh, PA). The study was conducted in accordance with the local legislation and institutional requirements.

## Author contributions

AS: Writing – original draft, Investigation. JJ: Investigation, Writing – review & editing. MV: Investigation, Writing – review & editing. HG: Data curation, Formal analysis, Writing – original draft. SL: Formal analysis, Methodology, Writing – review & editing. JC: Investigation, Writing – review & editing. KS: Investigation, Writing – review & editing. RD: Investigation, Formal analysis, Writing – review & editing. ZC: Supervision, Writing – review & editing. JW: Supervision, Writing – review & editing, Conceptualization, Funding acquisition, Project administration, Writing – original draft.

## References

[1] Bray F Ferlay J Soerjomataram I Siegel RL Torre LA Jemal A. Global cancer statistics 2018: GLOBOCAN estimates of incidence and mortality worldwide for 36 cancers in 185 countries. CA: A Cancer J Clin. (2018) 68:394–424. doi: 10.3322/caac.21492 30207593

[2] Cramer JD Burtness B Le QT Ferris RL. The changing therapeutic landscape of head and neck cancer. Nat Rev Clin Oncol. (2019) 16:669–83. doi: 10.1038/s41571-019-0227-z 31189965

[3] Du E Mazul AL Farquhar D Brennan P Anantharaman D Abedi-Ardekani B. Long-term survival in head and neck cancer: impact of site, stage, smoking, and human papillomavirus status. Laryngoscope. (2019) 129:2506–13. doi: 10.1002/lary.27807 PMC6907689 30637762

[4] Liang C Marsit CJ McClean MD Nelson HH Christensen BC Haddad RI. Biomarkers of HPV in head and neck squamous cell carcinoma. Cancer Res. (2012) 72:5004–13. doi: 10.1158/0008-5472.CAN-11-3277 PMC3463756 22991304

[5] Wang Z Xia RH Ye DX Li J. Human papillomavirus 16 infection and TP53 mutation: two distinct pathogeneses for oropharyngeal squamous cell carcinoma in an eastern chinese population. PloS One. (2016) 11:e0164491. doi: 10.1371/journal.pone.0164491 27749915 PMC5066983

[6] Leemans CR Braakhuis BJ Brakenhoff RH. The molecular biology of head and neck cancer. Nat Rev Cancer. (2011) 11:9–22. doi: 10.1038/nrc2982 21160525

[7] Johnson DE Burtness B Leemans CR Lui VWY Bauman JE Grandis JR. Head and neck squamous cell carcinoma. Nat Rev Dis Primers. (2020) 6:92. doi: 10.1038/s41572-020-00224-3 33243986 PMC7944998

[8] Gillison ML Shah KV. Human papillomavirus-associated head and neck squamous cell carcinoma: mounting evidence for an etiologic role for human papillomavirus in a subset of head and neck cancers. Curr Opin Oncol. (2001) 13:183–8. doi: 10.1097/00001622-200105000-00009 11307062

[9] Gillison ML Koch WM Capone RB Spafford M Westra WH Wu L. Evidence for a causal association between human papillomavirus and a subset of head and neck cancers. J Natl Cancer Institute. (2000) 92:709–20. doi: 10.1093/jnci/92.9.709 10793107

[10] Kamangar F Dores GM Anderson WF. Patterns of cancer incidence, mortality, and prevalence across five continents: defining priorities to reduce cancer disparities in different geographic regions of the world. J Clin Oncol: Off J Am Soc Clin Oncol. (2006) 24:2137–50. doi: 10.1200/JCO.2005.05.2308 16682732

[11] Chaturvedi AK Engels EA Pfeiffer RM Hernandez BY Xiao W Kim E. Human papillomavirus and rising oropharyngeal cancer incidence in the United States. J Clin Oncol: Off J Am Soc Clin Oncol. (2011) 29:4294–301. doi: 10.1200/JCO.2011.36.4596 PMC3221528 21969503

[12] Tezal M. Interaction between chronic inflammation and oral HPV infection in the etiology of head and neck cancers. Int J Otolaryngol. (2012) 2012:575242. doi: 10.1155/2012/575242 22518158 PMC3299260

[13] Canning M Guo G Yu M Myint C Groves MW Byrd JK. Heterogeneity of the head and neck squamous cell carcinoma immune landscape and its impact on immunotherapy. Front Cell Dev Biol. (2019) 7:52. doi: 10.3389/fcell.2019.00052 31024913 PMC6465325

[14] Cancer Genome Atlas N. Comprehensive genomic characterization of head and neck squamous cell carcinomas. Nature. (2015) 517:576–82. doi: 10.1038/nature14129 PMC4311405 25631445

[15] de Ruiter EJ Ooft ML Devriese LA Willems SM. The prognostic role of tumor infiltrating T-lymphocytes in squamous cell carcinoma of the head and neck: A systematic review and meta-analysis. Oncoimmunology. (2017) 6:e1356148. doi: 10.1080/2162402X.2017.1356148 29147608 PMC5674970

[16] Chen SMY Krinsky AL Woolaver RA Wang X Chen Z Wang JH. Tumor immune microenvironment in head and neck cancers. Mol Carcinog. (2020). 59 (7):766–74. doi: 10.1002/mc.23162 32017286 PMC7282929

[17] Bronte V Brandau S Chen SH Colombo MP Frey AB Greten TF. Recommendations for myeloid-derived suppressor cell nomenclature and characterization standards. Nat Commun. (2016) 7:12150. doi: 10.1038/ncomms12150 27381735 PMC4935811

[18] Chen SMY Li B Nicklawsky AG Krinsky AL Brunetti T Woolaver RA. Deletion of p53 and hyper-activation of PIK3CA in keratin-15(+) stem cells lead to the development of spontaneous squamous cell carcinoma. Int J Mol Sci. (2020) 21:6585. doi: 10.3390/ijms21186585 32916850 PMC7554792

[19] Chen SMY Popolizio V Woolaver RA Ge H Krinsky AL John J. Differential responses to immune checkpoint inhibitor dictated by pre-existing differential immune profiles in squamous cell carcinomas caused by same initial oncogenic drivers. J Exp Clin Cancer Res. (2022) 41:123. doi: 10.1186/s13046-022-02337-x 35366939 PMC8976353

[20] Strait AA Woolaver RA Hall SC Young CD Karam SD Jimeno A. Distinct immune microenvironment profiles of therapeutic responders emerge in combined TGFbeta/PD-L1 blockade-treated squamous cell carcinoma. Commun Biol. (2021) 4:1005. doi: 10.1038/s42003-021-02522-2 34433873 PMC8387430

[21] Lechner A Schlosser H Rothschild SI Thelen M Reuter S Zentis P. Characterization of tumor-associated T-lymphocyte subsets and immune checkpoint molecules in head and neck squamous cell carcinoma. Oncotarget. (2017) 8:44418–33. doi: 10.18632/oncotarget.17901 PMC5546490 28574843

[22] Schoenfeld JD Gjini E Rodig SJ Tishler RB Rawal B Catalano PJ. Evaluating the PD-1 axis and immune effector cell infiltration in oropharyngeal squamous cell carcinoma. Int J Radiat Oncol Biol Phys. (2018) 102:137–45. doi: 10.1016/j.ijrobp.2018.05.002 29960819

[23] Cingolani P Platts A Wang le L Coon M Nguyen T Wang L. A program for annotating and predicting the effects of single nucleotide polymorphisms, SnpEff: SNPs in the genome of Drosophila melanogaster strain w1118; iso-2; iso-3. Fly (Austin). (2012) 6:80–92. doi: 10.4161/fly.19695 22728672 PMC3679285

[24] Ge H Ferris RL Wang JH. Cetuximab responses in patients with HNSCC correlate to clonal expansion feature of peripheral and tumor-infiltrating T cells with top T-cell receptor clonotypes. Clin Cancer Res. (2023) 29:647–58. doi: 10.1158/1078-0432.CCR-22-2355 PMC9898159 36315045

[25] Vermeer DW Coppock JD Zeng E Lee KM Spanos WC Onken MD. Metastatic model of HPV+ oropharyngeal squamous cell carcinoma demonstrates heterogeneity in tumor metastasis. Oncotarget. (2016) 7:24194–207. doi: 10.18632/oncotarget.8254 PMC5029694 27013584

[26] De Groot R Van Loenen MM Guislain A Nicolet BP Freen-Van Heeren JJ Verhagen O. Polyfunctional tumor-reactive T cells are effectively expanded from non-small cell lung cancers, and correlate with an immune-engaged T cell profile. Oncoimmunology. (2019) 8:e1648170. doi: 10.1080/2162402X.2019.1648170 31646094 PMC6791436

[27] Egelston CA Avalos C Tu TY Simons DL Jimenez G Jung JY. Human breast tumor-infiltrating CD8(+) T cells retain polyfunctionality despite PD-1 expression. Nat Commun. (2018) 9:4297. doi: 10.1038/s41467-018-06653-9 30327458 PMC6191461

[28] Philip M Fairchild L Sun L Horste EL Camara S Shakiba M. Chromatin states define tumour-specific T cell dysfunction and reprogramming. Nature. (2017) 545:452–6. doi: 10.1038/nature22367 PMC5693219 28514453

[29] Priyadharshini B Welsh RM Greiner DL Gerstein RM Brehm MA. Maturation-dependent licensing of naive T cells for rapid TNF production. PloS One. (2010) 5:e15038. doi: 10.1371/journal.pone.0015038 21124839 PMC2991336

[30] Damuzzo V Pinton L Desantis G Solito S Marigo I Bronte V. Complexity and challenges in defining myeloid-derived suppressor cells. Cytometry B Clin Cytom. (2015) 88:77–91. doi: 10.1002/cyto.b.21206 25504825 PMC4405078

[31] Bassing CH Swat W Alt FW. The mechanism and regulation of chromosomal V(D)J recombination. Cell. (2002) 109 Suppl:S45–55. doi: 10.1016/S0092-8674(02)00675-X 11983152

[32] Jung D Alt FW. Unraveling V(D)J recombination; insights into gene regulation. Cell. (2004) 116:299–311. doi: 10.1016/S0092-8674(04)00039-X 14744439

[33] Poncette L Bluhm J Blankenstein T. The role of CD4 T cells in rejection of solid tumors. Curr Opin Immunol. (2022) 74:18–24. doi: 10.1016/j.coi.2021.09.005 34619457 PMC8933281

[34] Ben Khelil M Godet Y Abdeljaoued S Borg C Adotevi O Loyon R. Harnessing antitumor CD4(+) T cells for cancer immunotherapy. Cancers (Basel). (2022) 14:260. doi: 10.3390/cancers14010260 35008422 PMC8750687

[35] Li T Wu B Yang T Zhang L Jin K. The outstanding antitumor capacity of CD4(+) T helper lymphocytes. Biochim Biophys Acta Rev Cancer. (2020) 1874:188439. doi: 10.1016/j.bbcan.2020.188439 32980465

[36] Kravtsov DS Erbe AK Sondel PM Rakhmilevich AL. Roles of CD4+ T cells as mediators of antitumor immunity. Front Immunol. (2022) 13:972021. doi: 10.3389/fimmu.2022.972021 36159781 PMC9500154

[37] Borsetto D Tomasoni M Payne K Polesel J Deganello A Bossi P. Prognostic significance of CD4+ and CD8+ Tumor-infiltrating lymphocytes in head and neck squamous cell carcinoma: A meta-analysis. Cancers (Basel). (2021) 13:781. doi: 10.3390/cancers13040781 33668519 PMC7918220

[38] Xiang Y Gong M Deng Y Wang H Ye D. T cell effects and mechanisms in immunotherapy of head and neck tumors. Cell Commun Signal. (2023) 21:49. doi: 10.1186/s12964-023-01070-y 36872320 PMC9985928

[39] Wang JH. Why the outcome of anti-tumor immune responses is heterogeneous: A novel idea in the context of immunological heterogeneity in cancers. Bioessays. (2020) 42:e2000024. doi: 10.1002/bies.202000024 32767371 PMC7546576

[40] Woolaver RA Wang X Krinsky AL Waschke BC Chen SMY Popolizio V. Differences in TCR repertoire and T cell activation underlie the divergent outcomes of antitumor immune responses in tumor-eradicating versus tumor-progressing hosts. J Immunother Cancer. (2021) 9. doi: 10.1136/jitc-2020-001615 PMC7797305 33414263

[41] Chen Z John J Wang JH. Why responses to immune checkpoint inhibitors are heterogeneous in head and neck cancers: Contributions from tumor-intrinsic and host-intrinsic factors. Front Oncol. (2022) 12:995434. doi: 10.3389/fonc.2022.995434 36330485 PMC9623029

[42] John J Woolaver RA Popolizio V Chen SMY Ge H Krinsky AL. Divergent outcomes of anti-PD-L1 treatment coupled with host-intrinsic differences in TCR repertoire and distinct T cell activation states in responding versus non-responding tumors. Front Immunol. (2022) 13:992630. doi: 10.3389/fimmu.2022.992630 36330507 PMC9624473

[43] Hardaker EL Sanseviero E Karmokar A Taylor D Milo M Michaloglou C. The ATR inhibitor ceralasertib potentiates cancer checkpoint immunotherapy by regulating the tumor microenvironment. Nat Commun. (2024) 15:1700. doi: 10.1038/s41467-024-45996-4 38402224 PMC10894296

[44] Wu FL Nolan K Strait AA Bian L Nguyen KA Wang JH. Macrophages promote growth of squamous cancer independent of T cells. J Dent Res. (2019) 98:896–903. doi: 10.1177/0022034519854734 31189369 PMC6616122

[45] Boutilier AJ Elsawa SF. Macrophage polarization states in the tumor microenvironment. Int J Mol Sci. (2021) 22:6995. doi: 10.3390/ijms22136995 34209703 PMC8268869

[46] Costa NL Valadares MC Souza PP Mendonça EF Oliveira JC Silva TA. Tumor-associated macrophages and the profile of inflammatory cytokines in oral squamous cell carcinoma. Oral Oncol. (2013) 49:216–23. doi: 10.1016/j.oraloncology.2012.09.012 23089461

[47] Li B Ren M Zhou X Han Q Cheng L. Targeting tumor-associated macrophages in head and neck squamous cell carcinoma. Oral Oncol. (2020) 106:104723. doi: 10.1016/j.oraloncology.2020.104723 32315971

[48] Zverinova S Guryev V. Variant calling: Considerations, practices, and developments. Hum Mutat. (2022) 43:976–85. doi: 10.1002/humu.24311 PMC9545713 34882898

[49] Lefouili M Nam K. The evaluation of Bcftools mpileup and GATK HaplotypeCaller for variant calling in non-human species. Sci Rep. (2022) 12:11331. doi: 10.1038/s41598-022-15563-2 35790846 PMC9256665

[50] Cukalac T Chadderton J Handel A Doherty PC Turner SJ Thomas PG. Reproducible selection of high avidity CD8+ T-cell clones following secondary acute virus infection. Proc Natl Acad Sci United States America. (2014) 111:1485–90. doi: 10.1073/pnas.1323736111 PMC3910643 24474775

[51] Wellenstein MD de Visser KE. Cancer-cell-intrinsic mechanisms shaping the tumor immune landscape. Immunity. (2018) 48:399–416. doi: 10.1016/j.immuni.2018.03.004 29562192

[52] Ghorani E Swanton C Quezada SA. Cancer cell-intrinsic mechanisms driving acquired immune tolerance. Immunity. (2023) 56:2270–95. doi: 10.1016/j.immuni.2023.09.004 37820584

