# Supplementary material for: Differential tumor immune microenvironment coupled with tumor progression or tumor eradication in HPV-antigen expressing squamous cell carcinoma (SCC) models
Source: Front Immunol. 2024 Jul 11;15:1405318. doi: 10.3389/fimmu.2024.1405318 (PMC11269233; doi:10.3389/fimmu.2024.1405318)
Supplement: Supplementary file 1 [file DataSheet_1.pdf]

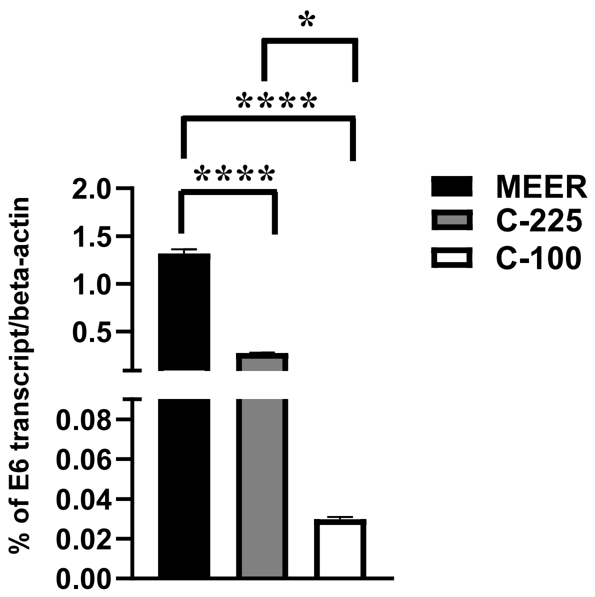

Supplemental Figure 1. Real-time PCR analysis of E6 transcripts in different tumor cell lines. Quantification of real-time PCR data from duplicates of one representative experiment using MEER, C-225, C-100 tumor cell lines. Statistical significance was calculated with one-way ANOVA; *p<0.05, **** p<0.0001.

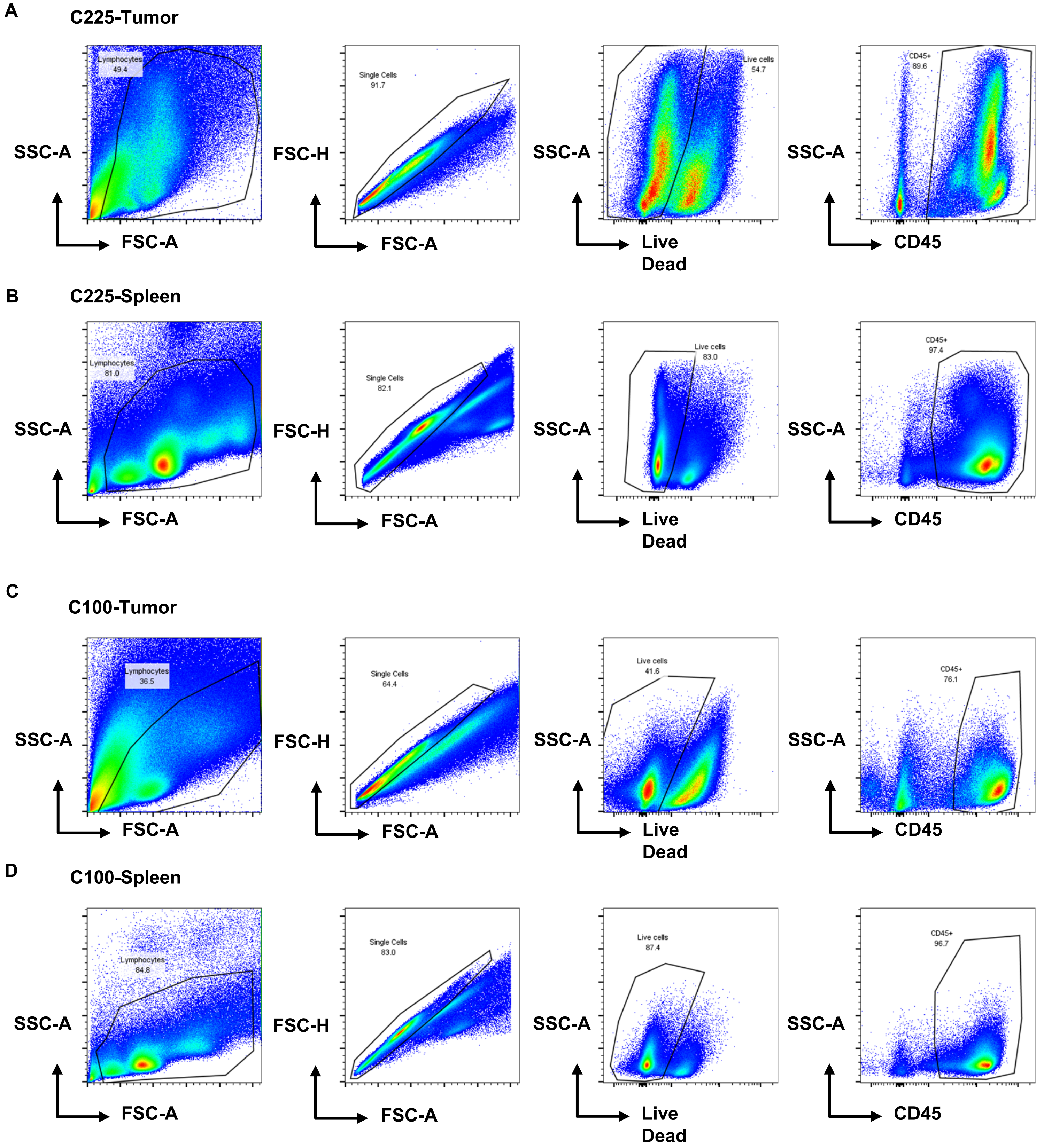

Supplemental Figure 2. Gating strategy for flow cytometry analysis. Single cell suspension was generated from tumor or spleen samples and analyzed by flow cytometry using the sequential gating as indicated.

Supplemental Figure 2

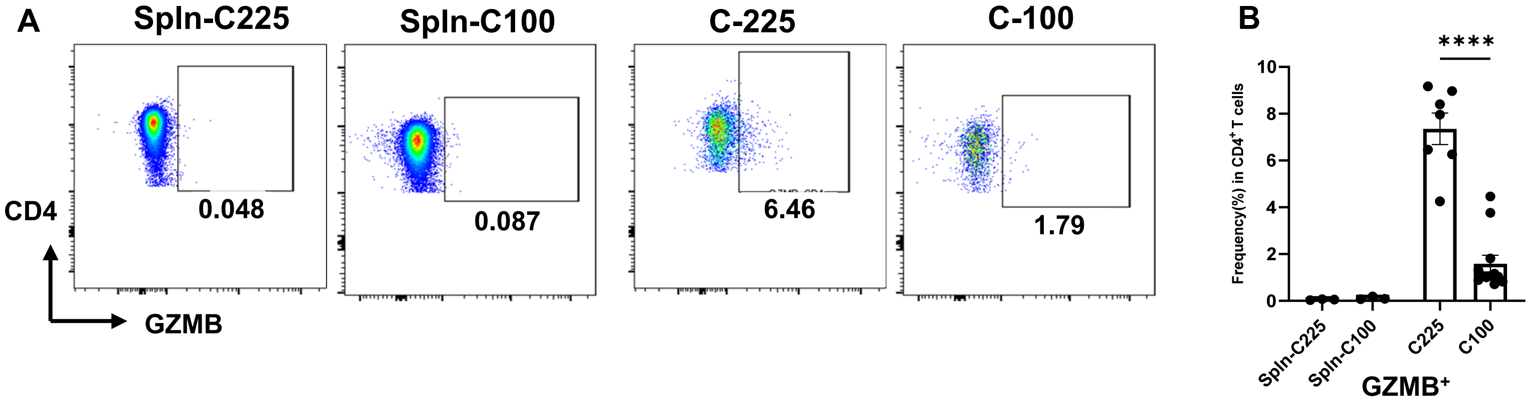

Supplemental Figure 3. Expression of GZMB in CD4 T cells. **(A)** Representative flow plots of CD4 T cells expressing GZMB. **(B)** Quantification of the percentage of CD4 T cells expressing GZMB in different groups. n=7 for C-225; n=12 for C-100. Statistical significance was calculated with an unpaired *t*-test; **** $p<0.0001$.

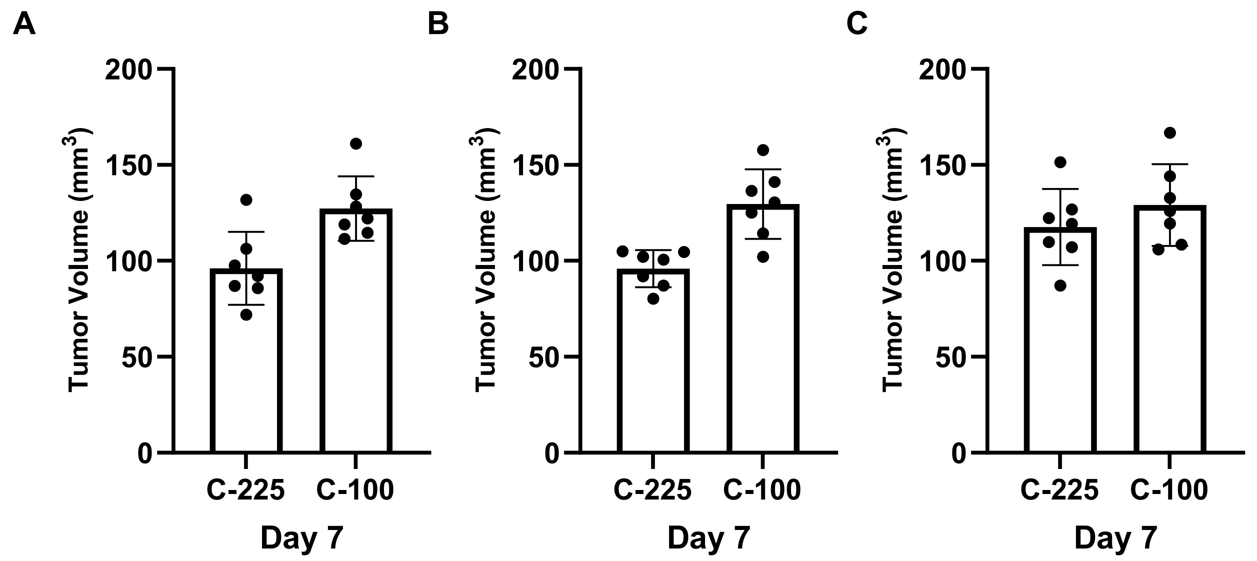

Supplemental Figure 4. Comparison of tumor volume of C-225 and C-100 on day-7**.** Tumor cells (2×10^6) were inoculated at the flank region of WT B6 mice (n=7 for C-100; n=7 for C-225). Panel (A), (B) & (C) represent three different cohorts.

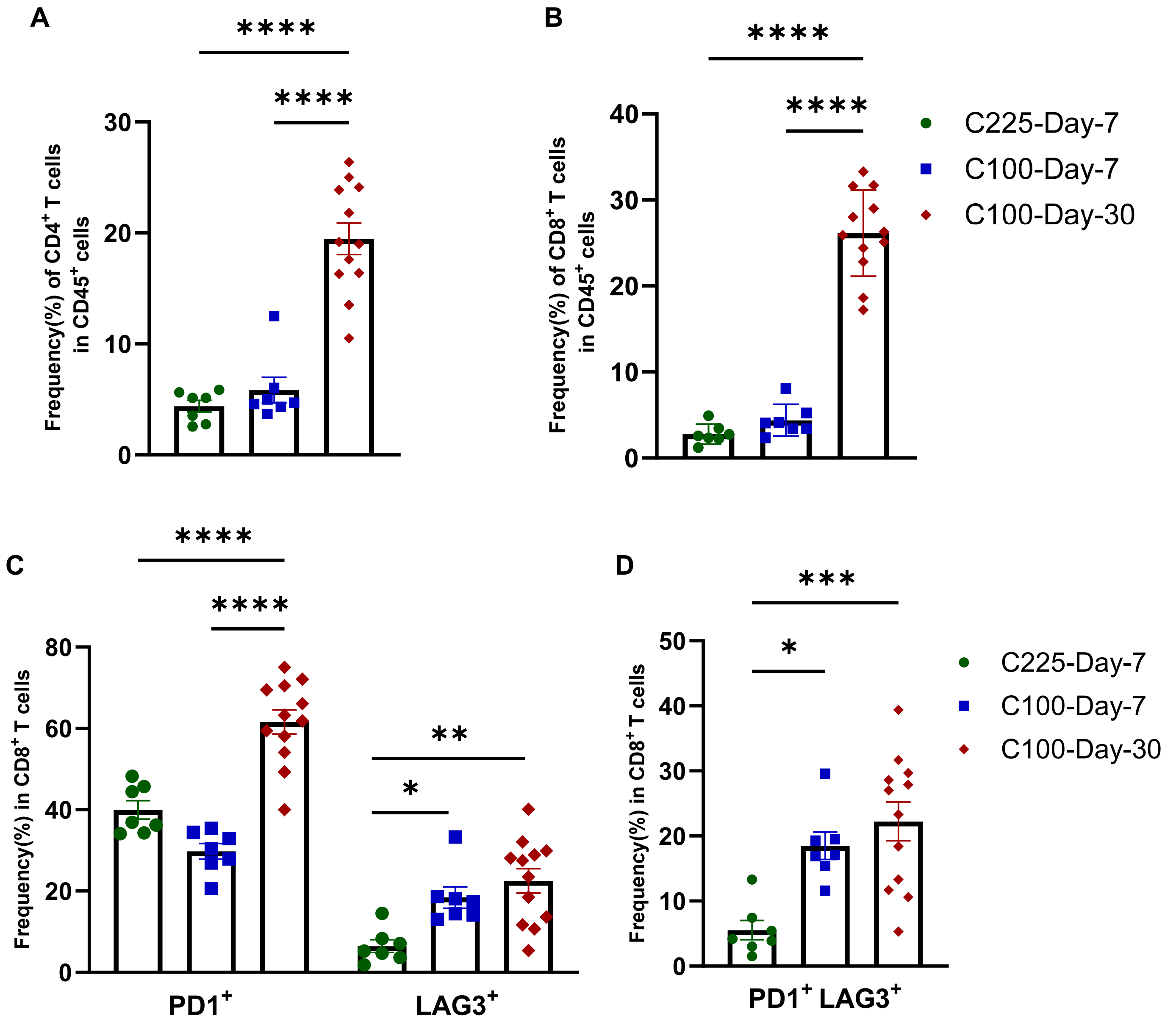

Supplemental Figure 5. Immune profiling of CD4 and CD8 TILs. Tumor cells (2×10^6) were inoculated into WT B6 mice at the flank region, and tumors were harvested from mice bearing C-225 on day-7 (C-225-day-7) or C-100 on day-7 (C-100-day-7) and day-30 (C-100-day-30). Flow cytometry analysis was performed for CD4 and CD8 TILs from C-225-day-7 (n=7), C-100-day-7 (n=7) and C-100-day-30 (n=12). **(A-B)** Representative graph shows quantification of the percentage of $CD4^+$ **(A)** and $CD8^+$ **(B)** T cells in $CD45^+$ population of C-225-day-7, C-100-day-7 or C-100-day-30 tumors. **(C-D)** Quantification of the percentage of CD8 TILs expressing different immune checkpoints in C-225-day-7, C-100-day-7 or C-100-day-30 tumors including PD-1 or LAG-3 **(C)** or both PD-1 and LAG-3 **(D).** Statistical significance was calculated with One-way ANOVA; $^*P<0.05$, $^{**}P<0.01$, $^{***}P<0.001$, $^{****}P<0.0001$.

Supplemental Figure 5

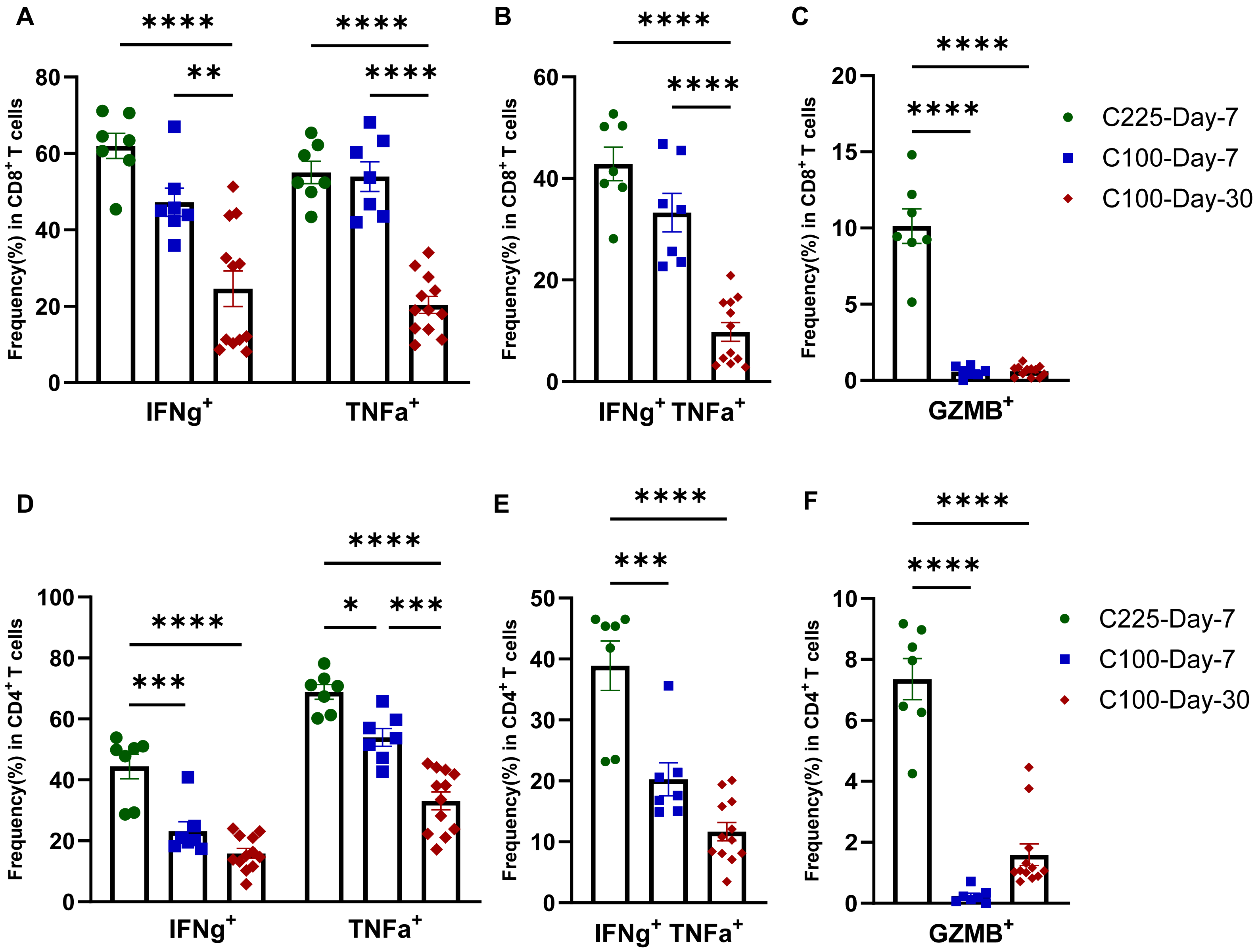

Supplemental Figure 6. Effector functions of CD8 and CD4 TILs. Flow cytometry analysis was performed to examine effector molecule expression on CD8 and CD4 TILs from C-225-day-7 (n=7), C-100-day-7 (n=7), and C-100-day-30 (n=12). **(A-C)** Representative graph shows quantification of the CD8 TILs expressing IFN-γ or TNF-α **(A)**, both IFN-γ^+ and TNF-α^+ **(B)**, or GZMB **(C)** in CD8 TILs. **(D-E)** Representative graph shows the percentage of CD4 TILs expressing IFN-γ or TNF-α **(D)**, both IFN-γ^+ and TNF-α^+ **(E)**, or GZMB **(F)** in CD4 TILs. Statistical significance was calculated with a One-way ANOVA; $^*P < 0.05$, $^{**}P < 0.01$, $^{***}P < 0.001$, $^{****}P < 0.0001$.

Supplemental Figure 6

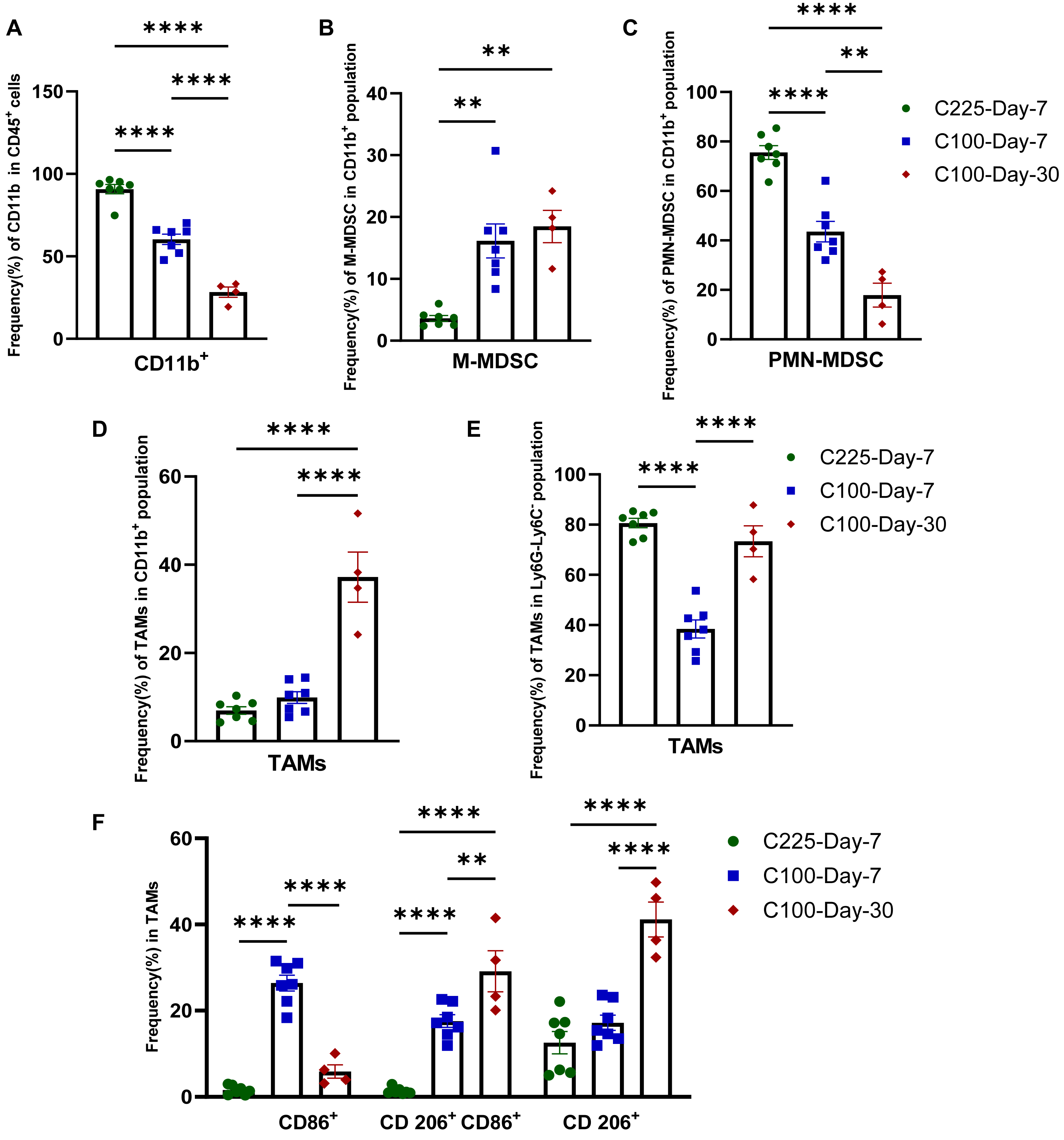

Supplemental Figure 7. Immune profiling of tumor-infiltrating myeloid cells. Flow cytometry analysis was performed as described in Supplementary Figure 5. **(A)** Representative graph shows the percentage of $CD11b^+$ population within the $CD45^+$ population in C-225-day-7 (n=7), C-100-day-7 (n=7), and C-100-day-30 (n=4) tumors. **(B-C)** Quantification of the percentage of M-MDSC **(B)** or PMN-MDSC **(C)** within $CD11b^+$ population. **(D-E)** Quantification of the percentage of TAMs ($F4/80^+$) within $CD11b^+$ **(D)** or $Ly6C^-Ly6G^-$ **(E)** population. **(F)** Quantification of the percentages of M1 ($CD86^+CD206^-$) and M2 ($CD86^-CD206^+$) TAMs. Statistical significance was calculated with One-way ANOVA; $^{**}P < 0.01$, $^{****} P<0.0001$.

Supplemental Figure 7

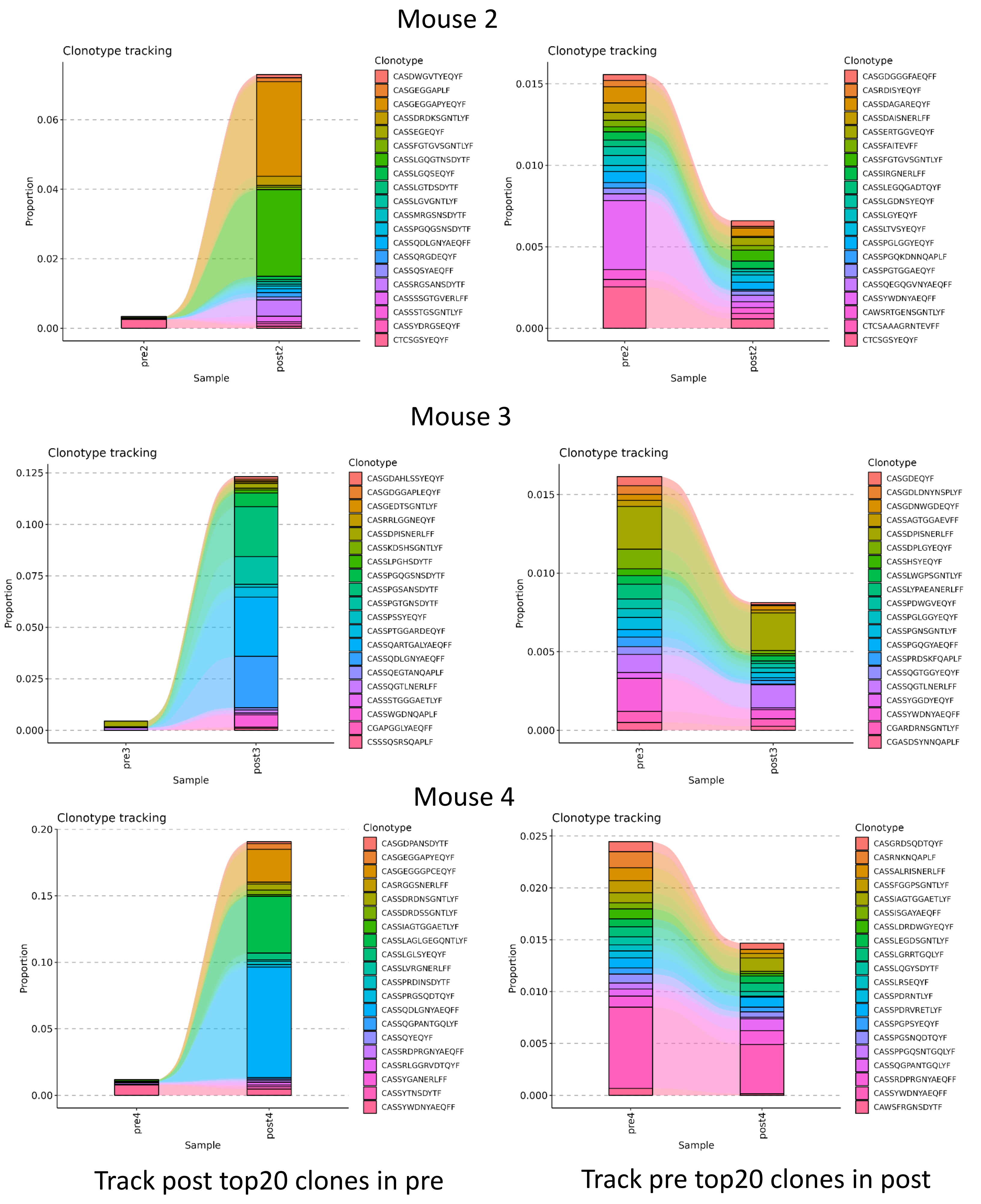

Supplemental Figure 8

Mouse 5

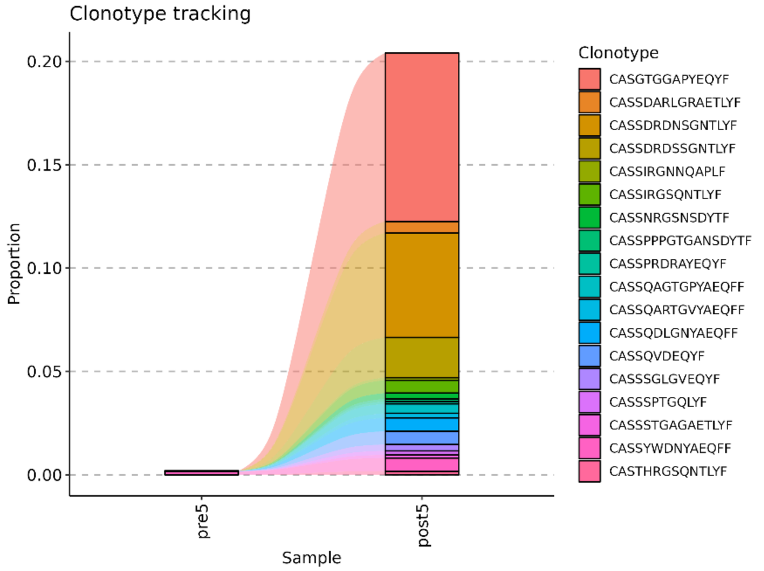

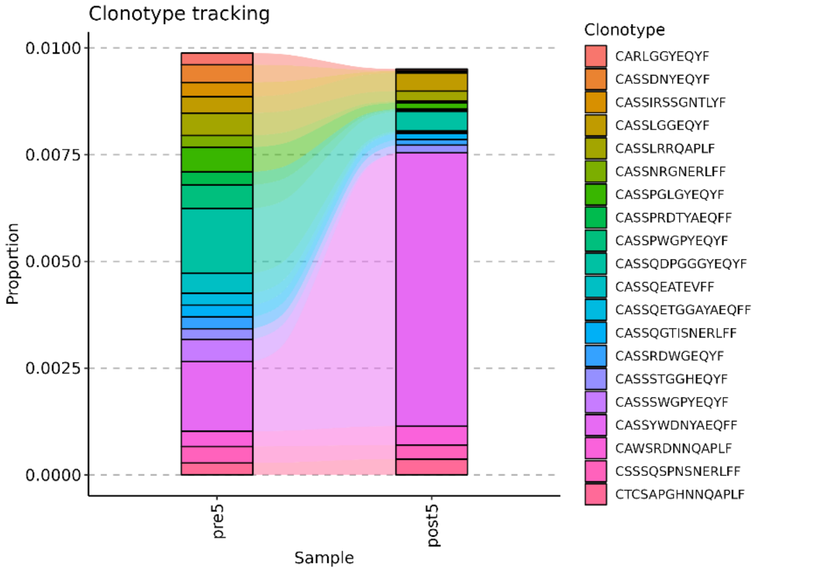

Mouse 6

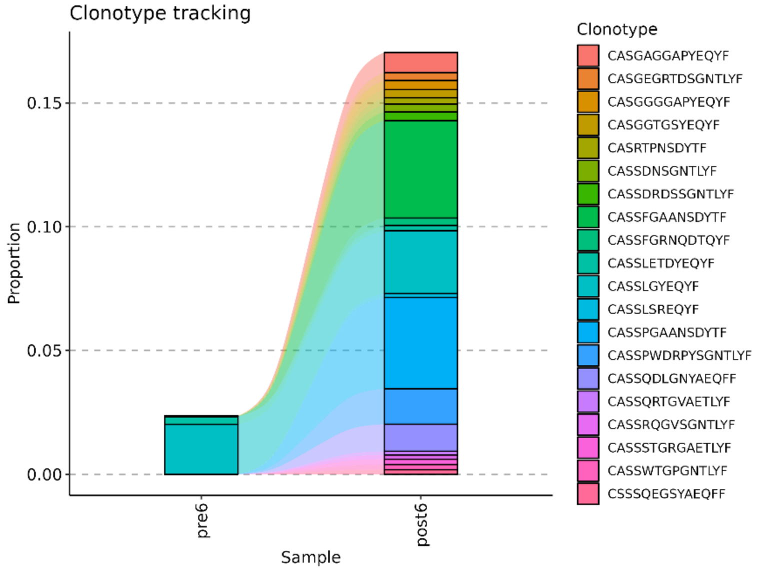

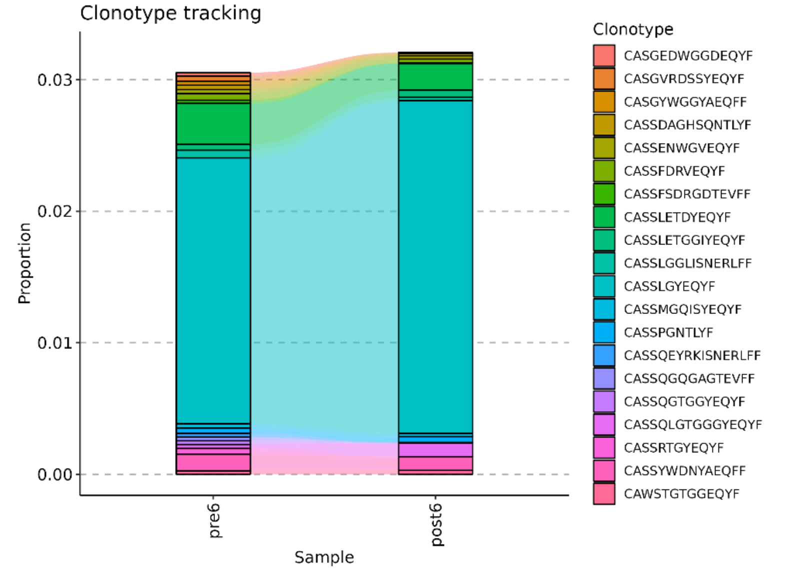

Mouse 7

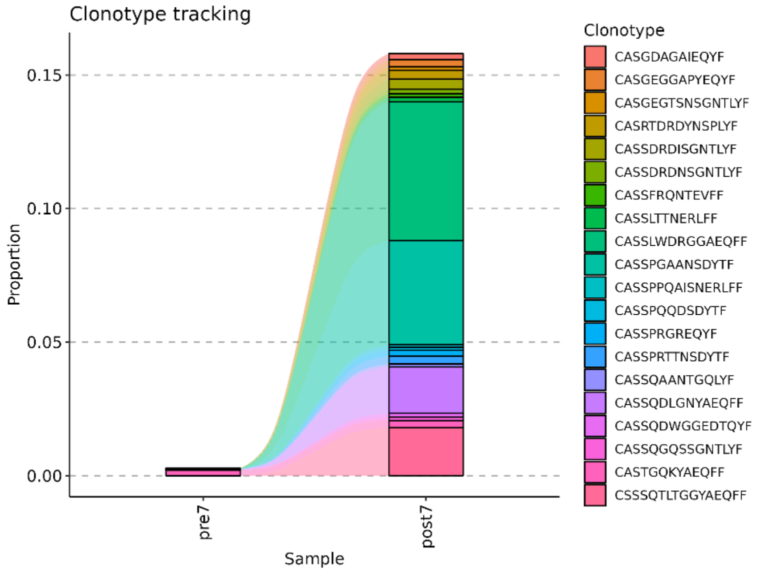

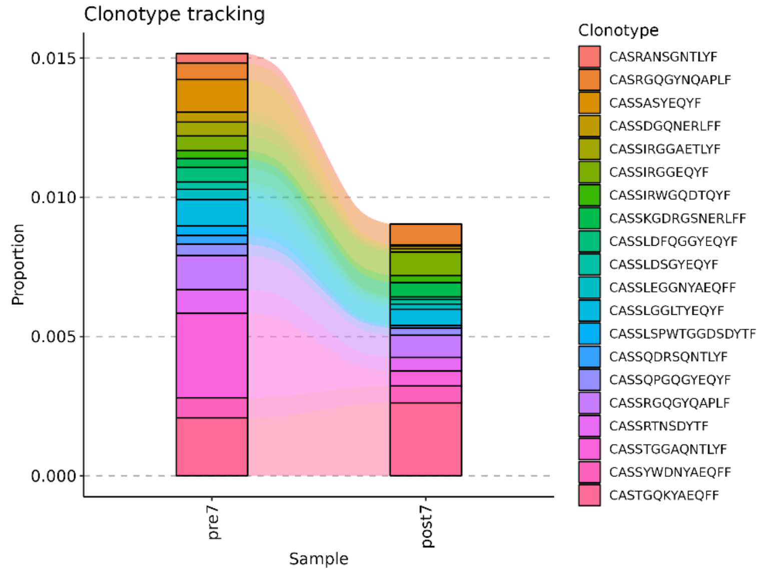

Track post top20 clones in pre

Track pre top20 clones in post

Supplemental Figure 8 continued

Supplemental Figure 8. Clonotype tracking between pre- vs. post- challenge samples for mouse 2 to mouse 7. Left: Clonotype tracking of post-challenge (post-2 to post-7) top-ranked 20 clones in corresponding pre-challenge samples (pre-2 to pre-7). **Right:** Clonotype tracking of pre-challenge (pre-2 to pre-7) top-ranked 20 clones in corresponding post-challenge sample (post-2 to post-7).

Antibody	Catalog	Clone	Company
Anti-HPV16 E7	SC-65711	NM2	Santa Cruz Biotechnologies
Anti-β-actin	sc-47778	C4	Santa Cruz Biotechnologies
HRP-anti-mouse-IgG	115-035-166	Poly	Jackson Immuno Research

FACS Antibody	Catalog	Clone	Company
BUV395 anti-CD45	564279	30-F11	BD Bioscience
BV 605 anti-CD8a	100744	53-6.7	BioLegend
BV 711 anti-CD8a	100748	53-6.7	BioLegend
BV 421 anti-CD4	100563	RM4-5	Biolegend
BV 711 anti-CD279 (PD-1)	135231	29F.1A12	Biolegend
PE anti-CD223 (LAG-3)	125207	C9B7W	BioLegend
PE anti-IFN-γ	12-7311-41	XMG1.2	eBioscience
APC anti-TNF-α	506308	mp6-xt22	Biolegend
FITC anti-Granzyme B	11-8898-82	NGZB	Invitrogen
Alexa Fluor 700 anti-CD11b	101222	M1/70	BioLegend
BV421 anti-Ly6C	128031	HK1.4	Biolegend
APC/Cy7 anti-Ly6G	127624	1A8	Biolegend
APC anti-F4/80	123115	BM8	Biolegend
FITC anti-CD86	105018	GL-1	BioLegend
PE/Cy7 anti-CD206	141719	C068C2	Biolegend

Supplemental Table 1

Supplemental Table 2: Fourteen samples sequenced by ImmunoSEQ assay

ID	Mouse	Tissue	Cells	Locus	Total templates	Productive templates	Total Rearrangements	Productive rearrangements
Pre1	C-225 Tumor rejected	Blood	PBMC	TCRB	88037	61785	67458	46603
Pre2	C-225 Tumor rejected	Blood	PBMC	TCRB	73598	51783	58299	40611
Pre3	C-225 Tumor rejected	Blood	PBMC	TCRB	68379	48069	52098	36021
Pre4	C-225 Tumor rejected	Blood	PBMC	TCRB	14940	10344	11649	7921
Pre5	C-225 Tumor rejected	Blood	PBMC	TCRB	52011	36221	40672	27991
Pre6	C-225 Tumor rejected	Blood	PBMC	TCRB	91241	65149	72550	50751
Pre7	C-225 Tumor rejected	Blood	PBMC	TCRB	63453	44247	50538	35076
Post1	C-225 Tumor bearing	Blood	PBMC	TCRB	157340	111393	115169	80280
Post2	C-225 Tumor bearing	Blood	PBMC	TCRB	81066	55985	57580	40133
Post3	C-225 Tumor bearing	Blood	PBMC	TCRB	145882	104165	97077	67696
Post4	C-225 Tumor bearing	Blood	PBMC	TCRB	18641	13295	11585	7676
Post5	C-225 Tumor bearing	Blood	PBMC	TCRB	73508	54484	45085	31124
Post6	C-225 Tumor bearing	Blood	PBMC	TCRB	28596	20240	18442	12477
Post7	C-225 Tumor bearing	Blood	PBMC	TCRB	63243	42821	39484	27355

Supplemental Table 3. Real-time PCR primers

**Real-time primers**			
**Gene**	**Primer Type**	**Primer sequences (5' to 3')**	**PCR Conditions**
**E6**	F<br>R	CAGGAGCGACCCAGAAAGTT<br>CAGCTGGGTTTCTCTACGTGT	94°C 5 min, 95°C 20s, 55°C 15s, 72°C 15s, 30 cycles, 72°C 10 min
**β-actin**	F<br>R	TGGAATCCTGTGGCATCCATGAAAC<br>TAAAACGCAGCTCAGTAACAGTCCG	94°C 5 min, 95°C 20s, 55°C 15s, 72°C 15s, 30 cycles, 72°C 10 min
